# Proceedings of the Canadian Society of Allergy and Clinical Immunology Annual Scientific Meeting 2017

**DOI:** 10.1186/s13223-018-0240-2

**Published:** 2018-03-29

## Allergic Rhinitis/Asthma

### A01 Trend in the prevalence of asthma and its symptoms amongst Mexican late adolescents: a study of 7 years apart

#### Tonantzin I. Bedolla-Pulido^1^, Tonatiuh R. Bedolla-Pulido^2^, Jaime Morales-Romero^3^, Grace A. López-Cota^4^, Martín Robles-Figueroa^4^, Eduardo Navarro-Lozano^1^, and Martín Bedolla-Barajas^2^

##### ^1^Centro Universitario de Ciencias de la Salud, Universidad de Guadalajara, Guadalajara, Jalisco, México; ^2^Servicio de Alergia e Inmunología Clínica, Hospital Civil de Guadalajara “Dr. Juan I. Menchaca”, Guadalajara, Jalisco, México; ^3^Instituto de Salud Pública, Universidad Veracruzana, Xalapa, Veracruz, México; ^4^Servicio de Medicina Interna, Hospital Civil de Guadalajara “Dr. Juan I. Menchaca”, Guadalajara, Jalisco, México

###### **Correspondence:** Tonantzin I. Bedolla-Pulido

*Allergy, Asthma & Clinical Immunology * 2018, **14(Suppl 1):**A01

**Background:** Several regions around the world continue to see increases in the prevalence of asthma and its symptoms; however, in our population the studies are scarce. On the other hand, the population of late adolescents has rarely been analyzed. Thus, this study was conducted to determine the trend of the prevalence of asthma and its symptoms in late adolescents over a period of 7 years.

**Methods:** Two cross-sectional population-based studies were conducted in 2009 and 2016 in Mexican adolescents, aged 15–18 years. Prevalence of asthma and its symptoms, allergic rhinitis and atopic dermatitis were compared between both studies. The data was obtained through the questionnaire proposed by The International Study of Asthma and Allergies in Childhood. An analysis for linear trend for proportions (Mantel–Haenszel) was performed.

**Results:** In 2009, 1600 adolescents were included, and in 2016, 1992 were included. In general, the presence of wheezing at any time during the previous year increased significantly between 2009 and 2016 (12.8% vs. 20.1% and 7.3% vs. 10.3%, p < 0.001 and 0.002, respectively). The prevalence of asthma increased from 7.8% in 2009 to 12.7% in 2016 (p < 0.001). In addition, the prevalence of allergic rhinitis (4.5% vs 9.0%) and atopic dermatitis (3.8% vs 5.2%) also suffered marked increases (p < 0.001 and 0.042, respectively).

**Conclusions:** The prevalence of asthma and some of its symptoms in late adolescents increased substantially in the previous 7 years.

### A02 Effect of exposure to dogs and cats over prevalence of allergic diseases amongst Mexican school-aged children

#### Tonatiuh R. Bedolla-Pulido^1^, Tonantzin I. Bedolla-Pulido^2^, Norma A. Pulido-Guillén^3^, Jaime Morales-Romero^4^, Carlos Meza-López^5^, and Martín Bedolla-Barajas^1^

##### ^1^Servicio de Alergia e Inmunología Clínica, Hospital Civil de Guadalajara “Dr. Juan I. Menchaca”, Guadalajara, Jalisco, México; ^2^Centro Universitario de Ciencias de la Salud, Universidad de Guadalajara, Guadalajara, Jalisco, México; ^3^Psicología Clínica. Guadalajara, Jalisco, México; ^4^Instituto de Salud Pública, Universidad Veracruzana, Xalapa, Veracruz, México; ^4^Servicio de Medicina Interna, Hospital Civil de Guadalajara “Dr. Juan I. Menchaca”, Guadalajara, Jalisco, México

###### **Correspondence:** Tonatiuh R. Bedolla-Pulido

*Allergy, Asthma & Clinical Immunology* 2018, **14(Suppl 1):**A02

**Background:** The association regarding the exposure to pets, especially cats and dogs, and the prevalence of allergic diseases is inconsistent. We analyzed the role that early exposure to dogs and cats plays in the prevalence of allergic diseases amongst school-aged children.

**Methods:** Through a cross-sectional study, we examined 756 children, aged 6–7; these candidates were selected through cluster sampling. We inquired about the exposure that these children had to dogs and cats, and whether these pets spent most of their time indoors or outdoors during the first year of the child’s life. In order to identify the prevalence of allergic diseases and their symptoms each child’s parent completed the *International Study of Asthma and Allergies in Childhood* questionnaire.

**Results:** Contact with outdoor dogs was associated to nocturnal coughing, odds ratio (OR) 0.64, with a confidence interval at 95% (CI 95%) 0.43–0.95 and with atopic dermatitis (OR: 0.39; CI 95%: 0.20 a 0.76); while outdoor contact with cats was associated to nocturnal coughing (OR: 0.51; CI 95%: 0.32 a 0.83) and the current symptoms of rhinitis (OR: 0.59; CI 95% 0.36 a 0.97). After carrying out the multivariate analyses, only exposure to dogs, both indoor and outdoor, was significantly associated to the prevalence of atopic dermatitis OR 0.40 (CI 95%: 0.20 a 0.79) and OR 0.38 (CI 95%: 0.18 a 0.83), respectively.

**Conclusions:** Our findings suggest that the exposure to dogs, whether they be indoor or outdoor pets, is associated to a decreased prevalence in atopic dermatitis.

### A03 Determining if asthma risk at 5 years can be predicted in early life using infant and preschool pulmonary function tests (PFTs)

#### Helen Cai^1,2^, Rachel Foong^1,3^, Aimée Dubeau^1^, Zihang Lu^1,4^, Vera Dai^1,4^, Krzysztof Kowalik^2,5^, Per Gustafsson^6^, Felix Ratjen^2^, Malcom R. Sears^7^ and Padmaja Subbarao^2,5^

##### ^1^Faculty of Applied Health Sciences, University of Waterloo, Waterloo, Ontario, Canada; ^2^Division of Respiratory Medicine and Program in Translational Medicine, SickKids Research Institute, and The Hospital for Sick Children, Toronto, Ontario, Canada; ^3^School of Physiotherapy & Exercise Science, Curtin University, Bentley, Western Australia, Australia; ^4^Dalla Lana School of Public Health, University of Toronto, Toronto, Canada; ^5^Department of Physiology, Faculty of Medicine, University of Toronto, Toronto, Ontario, Canada; ^6^Department of Pediatrics, Central Hospital, Skövde, Sweden; ^7^Department of Medicine, Faculty of Health Sciences, McMaster University, Hamilton, Ontario, Canada

###### **Correspondence:** Helen Cai

*Allergy, Asthma & Clinical Immunology* 2018, **14(Suppl 1):**A03

**Background:** Longitudinal assessments of lung function from birth throughout childhood are crucial to better elucidate the natural history of asthma’s disease process in early life. Multiple-breath washout (MBW) yields Lung Clearance Index (LCI) values, a sensitive measure of ventilation inhomogeneity, while exhaled fractional nitric oxide (FeNO) is a measure of airway inflammation shown to be elevated in asthmatics. Both techniques are non-invasive and require minimal cooperation, making them ideal pulmonary function tests (PFTs) to investigate asthma trajectory in pediatric populations. We aim to determine whether infant PFT can be used clinically as a predictive tool for asthma diagnosis in young children.

**Methods:** MBW and FeNO were measured in infants aged 2.5 months to 3 years in the Canadian Healthy Infant Longitudinal Development (CHILD) study’s Toronto general cohort. Both were standardized using a reference population to account for height-related changes. Asthma diagnosis and wheeze frequency were determined from 5 year follow-up clinical assessments. Comparisons of asthma and wheeze groups were made using nonparametric tests (Mann–Whitney and Kruskal–Wallis) and a logistic regression model explored associations between early life PFT and 5 year outcomes.

**Results:** LCI and FeNO in infancy were not significantly different between 5 year asthma status and wheeze frequency groups and neither variable contributed to the logistic regression model (p > 0.05). LCI at 3 years of age was significantly elevated in those destined to develop asthma at age 5 and in those with recurrent wheeze (p < 0.0001).

**Conclusions:** Three years but not infant lung function, is predictive of diagnosed asthma or recurrent wheeze at 5 years. This suggests that changes occurring in the first 3 years of life are critical to the development of lung dysfunction associated with recurrent wheeze or asthma diagnosis at age 5. Further investigation into the timing of this early lung development is required thus additional analysis is ongoing.

### A10 Relation bronchial asthma and parasitic (nematodes) infection in Egyptian children

#### Y. Habib^1^, M. Shaheen^2^, M. Zidan^2^, H. Gharraf^2^, A. Abd Elftah^3^

##### ^1^Alexandria Police Hospital, Alexandria, Egypt; ^2^Chest Department, Alexandria University, Alexandria, Egypt; ^3^Medical Research Institute, Alexandria University, Alexandria, Egypt

###### **Correspondence:** Y. Habib

*Allergy, Asthma & Clinical Immunology* 2018, **14(Suppl 1):**A10

**Background:** Geo-helminthic infections are among the many factors influencing the prevalence of asthma in developing countries from the tropics.

**Aims:** This work aims to study the relation between bronchial asthma and parasitic infestation in Egyptian children.

**Patients and methods:** A cross-section, analytical study design was chosen to perform this research on 100 school aged children. All children were interviewed and examined clinically and laboratory.

**Results:** 86% of patients with bronchial asthma lived in urban areas, while 64% of patients with parasitic infestation lived in rural areas. Statistically significantly negative correlations were found between blood level of IgE and FEV1% of predicted in patients with bronchial asthma as well as patients with parasitic infestation with r = − 0.381, − 0.325 at p = 0.006, 0.021 respectively. Inverse relationship was found between blood level of IgE and FEV1/FVC% in patients with parasitic infestation with r = − 0.358 with statistical significant difference at p = 0.011.

**Conclusions:** Statistically, significantly higher values of IgE were found in patients with parasitic infestation compared to patients with bronchial asthma. It was noted that patients with combined bronchial asthma and parasitic infestation demonstrated statistically significance higher values of IgE which suggest a possible synergistic effect of two diseases.

**Recommendation:** Improving personal and environmental hygiene, regular screening, and treatment and health education for children as regard parasitic infections is recommended.

### A13 Asthmatic release of lysophosphatidic acid mediates neurogenic bronchoconstriction through stimulation of the carotid body

#### Nicholas G. Jendzjowsky^1^, Arijit Roy^1^, Margaret M. Kelly^1,2^, Francis H.Y. Green^2^, and Richard J.A. Wilson^1^

##### ^1^Department of Physiology and Pharmacology, Cumming School of Medicine, University of Calgary, Calgary, Alberta, Canada; ^2^Department of Pathology and Laboratory Medicine, Cumming School of Medicine, University of Calgary, Calgary, Alberta, Canada

###### **Correspondence:** Nicholas G. Jendzjowsky

*Allergy, Asthma & Clinical Immunology* 2018, **14(Suppl 1):**A13

**Background:** The inflammatory mediator lysophosphatidic acid (LPA) increases during an asthmatic attack, LPA is a TRPV1 agonist, and TRPV1 is expressed in the carotid body. Given that carotid body stimulation causes bronchoconstriction, we hypothesized that LPA would trigger asthmatic bronchoconstriction through stimulation of the carotid body.

**Methods:** We tested the effects of LPA on carotid sinus nerve discharge in a novel perfused *en bloc* carotid body preparation and measured lung resistance in the ovalbumin sensitized (OVA) and naïve (N) anesthetized rat model of asthma. The carotid body response to LPA (5 µM) was tested along with the effects of TRPV1 (AMG9810, 10 µM) and LPA receptor (LPAr) blockade (BrP-LPA, 1.5 µM). RT-PCR was performed to probe for LPA receptors (LPAr) 1–4 in rat carotid body, petrosal ganglia and superior cervical ganglia. In anesthetized preparations, broncho-provocation was induced by nebulization of 100 µM bradykinin for 30 breaths in OVA and N rats and lung resistance was measured with a flexivent system. In order to associate LPA increase to carotid body stimulation, ELISA assessed arterial plasma LPA concentration before and after bradykinin mediated broncho-provocation.

**Results:** Carotid sinus nerve discharge increased with LPA, was reduced with BrP-LPA (p < 0.05) and BrP-LPA+AMG9810 (p < 0.05). LPAr 1, 3, 4 were expressed in carotid body, petrosal ganglia and superior cervical ganglia. Plasma LPA was increased in OVA compared to N prior to and following bradykinin (p < 0.05). Lung resistance induced with OVA exposure was reduced by carotid body denervation, vagotomy, TRPV1 blockade, LPAr blockade and combined LPAr+TRPV1 blockade (p < 0.05).

**Conclusions:** The carotid body senses LPA via TRPV1 *and* LPAr resulting in bronchoconstriction. Our data demonstrate an important component of asthmatic neurogenic bronchoconstriction mediated by the carotid body, linking inflammatory mediators and the induction of neurogenic bronchoconstriction.

### A14 Immune gene signatures in blood of patients with allergic rhinitis following nasal allergen challenge

#### Young Woong Kim^1,2,3^, Casey P. Shannon^3^, Amrit Singh^2,3,4^, Anne K. Ellis^5,6^, Helen Neighbour^7,8^, Mark Larché^7,8^, Scott J. Tebbutt^1,2,3,9^

##### ^1^Experimental Medicine, University of British Columbia, Vancouver, British Columbia, Canada; ^2^Centre for Heart Lung Innovation, St. Paul’s Hospital, Vancouver, British Columbia, Canada; ^3^Prevention of Organ Failure (PROOF) Centre of Excellence, Vancouver, British Columbia, Canada; ^4^Department of Pathology and Laboratory Medicine, University of British Columbia, Vancouver, British Columbia, Canada; ^5^Departments of Medicine and Biomedical & Molecular Science, Queen’s University, Kingston, Ontario, Canada; ^6^Allergy Research Unit, Kingston General Hospital, Kingston, Ontario, Canada; ^7^Department of Medicine, McMaster University, Hamilton, Ontario, Canada; ^8^Firestone Institute for Respiratory Health, McMaster University, Hamilton, Ontario, Canada; ^9^Department of Medicine (Division of Respiratory Medicine), University of British Columbia, Vancouver, British Columbia, Canada

###### **Correspondence:** Young Woong Kim

*Allergy, Asthma & Clinical Immunology* 2018, **14(Suppl 1):**A14

**Background:** Allergic rhinitis (AR) is not only a local inflammatory disease, but also a systemic disease. To better understand the systemic immune aspects of the pathophysiology, we investigated canonical immune gene expression in peripheral blood (in which immune deviations associated with allergy have previously been reported) collected from research participants undergoing a nasal allergen challenge (NAC). The NAC model allows investigation of AR pathophysiology with biological sampling.

**Methods:** Nine patients with AR secondary to cat allergy underwent NAC with standardized cat allergen. Clinical symptom changes after the allergen challenge were assessed by Total Nasal Symptom Score (TNSS) and Peak Nasal Inspiratory Flow (PNIF). Peripheral blood was collected at baseline and post NAC (1, 2 and 6 h). Canonical immune genes (730) were profiled from PAXgene blood lysates using a NanoString nCounter assay. Statistical analyses of complete blood count (CBC) data and gene expression data were performed using the R statistical computing program.

**Results:** TNSS and PNIF changes confirmed a significant AR response following the NAC. This was accompanied by significant changes in immune cell frequencies: eosinophils (1 and 6 h) decreased, while neutrophils (1 and 2 h), lymphocytes (6 h), monocytes (2 and 6 h), and total leukocytes (2 and 6 h) significantly (p < 0.05) increased after the allergen challenge compared to baseline. After statistical (FDR < 0.1) and fold change (> ± 1.2) thresholds were applied, 120 immune genes were retained and were clustered into seven distinct gene signatures that demonstrated different gene expression patterns following the allergen challenge.

**Conclusions:** Systemic immune responses following allergen challenge were demonstrated via changes in blood cell counts and immune gene expression. These changes may be useful to examine efficacy or mechanism of action of AR treatments when combined with the NAC model.

### A15 Quantification of specific IgE & IgG against pigeon allergens in asthmatic patients: an Indian study

#### Anil K. Mavi^1^, Raj Kumar^2^, Deepak Kumar^3^

##### ^1^Department of Respiratory Allergy, Vallabhbhai Patel Chest Institute University of Delhi, New Delhi, Delhi, India; ^2^Asthma & Applied Immunology, Vallabhbhai Patel Chest Institute University of Delhi, New Delhi, Delhi, India; ^3^Department of Pulmonary Medicine, Vallabhbhai Patel Chest Institute University of Delhi, New Delhi, Delhi, India

###### **Correspondence:** Anil K. Mavi

*Allergy, Asthma & Clinical Immunology* 2018, **14(Suppl 1):**A15

**Background:** Detecting serum antibody against inhaled antigens is an important diagnostic adjunct for hypersensitivity in asthmatic patients. In India, people tend to keep pigeons as an ancient and respected custom or for religious purposes. We aim to study the quantification of specific IgE & IgG against pigeon allergens in the blood serum of asthmatic patients.

**Methods:** A total of 200 asthmatic patients diagnosed as per GINA guideline (15–58 years old) were enrolled for the study. All subjects underwent routine investigations and skin prick testing against common aero-allergens and pigeon allergens. All patients underwent serum estimation of specific IgE and IgG against pigeon droppings and feathers with the help of ImmunoCAP100.

**Results:** Out of the total 200 subjects (81 males and 119 females) history of exposure to pigeons was present in 108 patients (54%). Out of the 200 patients, 68 (34%) had positive SPT to ≤ 3 common aeroallergens and 40 (20%) SPT positive ≥ 3 common aeroallergens. In SPT positivity of pigeon allergens feathers and droppings, 4 patients (2%) and 14 (7%) patients were positive respectively. The mean value of specific IgE & IgG of SPT against droppings was 1.4 KUA/L and 28.12 MgA/L and feathers were 0.0075 KUA/L and 9.625 MgA/L respectively. Specific IgE of pigeon exposure against droppings and feather was higher in patients exposed to pigeon droppings and feathers with their mean values 0.31KUA/L (p = 0.013) and 0.093 KUA/L (p = 0.001) in comparison to non-exposed groups with their mean values droppings 0.15 KUA/L and feathers 0.041 KUA/L. Specific IgG of patients exposed group against droppings and feather was higher 25.52 MgA/L (p = 0.009) and 5.61 MgA/L (p = 0.064) in comparison to non-exposed groups dropping and feather 20.61MgA/L and 4.60 MgA/L respectively.

**Conclusions:** Specific IgE and IgG against pigeon droppings and feathers exposed patients was high in comparison to non-exposed asthmatic patients.

### A16 Assessing Canadian children’s exposure to phthalates and polycyclic aromatic hydrocarbons (PAHs)

#### Garthika Navaranjan^1^, Edem A. Afenu^2^, Miriam L. Diamond^1,3^, Jeffrey R. Brook^1,4^

##### ^1^Dalla Lana School of Public Health, University of Toronto, Toronto, Ontario, Canada; ^2^Department of Biochemistry, University of Toronto, Toronto, Ontario, Canada; ^3^Department of Earth Sciences, University of Toronto, Toronto, Ontario, Canada; ^4^Environment Canada, Toronto, Ontario, Canada

###### **Correspondence:** Garthika Navaranjan

*Allergy, Asthma & Clinical Immunology* 2018, **14(Suppl 1):**A16

**Background:** Exposures to chemicals in the indoor environment, where children spend much of their early life, are important to examine in the development of childhood asthma and allergic disease. However, little has been done to characterize children’s exposures to these chemicals, including phthalates and polycyclic aromatic hydrocarbons (PAHs), which have widespread use or release into the household environment. This analysis will quantify Canadian children’s exposure to phthalates and PAHs as a first step in examining environmental triggers of childhood asthma and allergic disease onset.

**Methods:** Infants were recruited in Vancouver as part of a pilot for the Canadian Healthy Infant Longitudinal Development study. The pilot involved in-home visits with questionnaires administered, and collection of infant urine, floor dust and surface wipes of window films from infant bedrooms and most used room (MUR). Concentrations of six phthalates and 16 PAHs were statistically analyzed for differences across room type using paired *t* test and correlations between dust and window wipes using Spearman’s correlation.

**Results:** Forty infants were recruited as part of the pilot. The highest concentration was observed for phthalates DEHP in dust (geometric mean (GM)_dus_t:146.74 ng/mg) and window films (GM_window_:837.61 ng/wipe) and DiNP (GM_dust_:153.26 ng/mg; GM_window_:692.51 ng/wipe). Concentrations of PAHs were lower and ranged from 0.08 to 0.73 ng/mg dust and 0.6 to 1.35 ng/wipe. GM concentrations of phthalates and PAHs appeared higher in bedrooms than MUR, but was only statistically significant for DEHP (p = 0.03) in dust, and BnBP (p = 0.02) and benzo[b] fluoranthene (p = 0.01) in window wipes. A significant positive correlation was only found between BzBP in dust and window wipes in bedroom (r = 0.34; p = 0.04) and MUR (r = 0.39; p = 0.02).

**Conclusions:** Dust and window film samples showed phthalate concentrations an order of magnitude higher than those of PAH, with the highest concentrations found for DEHP and DiNP. Further work will explore the association between infant’s household phthalate and PAH exposure, and development of asthma and allergies.

### A17 The synergistic role of human rhinovirus and TGFβ1 in the pathogenesis of airway remodeling in asthma

#### Diana Pham^1,2^, Cora Kooi^1,2^, David Proud^2^, Richard Leigh^1,2^

##### ^1^Department of Medicine, University of Calgary, Calgary, Alberta, Canada; ^2^Department of Physiology and Pharmacology, University of Calgary, Calgary, Alberta, Canada

###### **Correspondence:** Diana Pham

*Allergy, Asthma & Clinical Immunology* 2018, **14(Suppl 1):**A17

**Background:** Airway remodeling (AR) is a characteristic feature of asthma that develops in early childhood, but is not present in infants. This suggests that AR is not congenital, but rather develops in response to some stimulus in early life. Studies have suggested that recurrent human rhinovirus (HRV) infections may have a role in airway remodeling. Structural cells called myofibroblasts are more abundant in asthmatic airways, though their origin is unknown. These myofibroblasts contribute to AR by depositing extracellular matrix proteins in the lamina reticularis and subepithelial region, resulting in airway wall thickening and airflow limitation. In this study, we sought to investigate whether HRV-infected epithelial cell supernatants could induce fibroblasts to develop phenotypic characteristics of myofibroblasts.

**Methods:** Human bronchial epithelial cells (HBE) and fibroblasts (HBF) were isolated from non-transplanted normal human lungs. HBE cells were grown for 14 days until confluent and then infected with HRV-16 in basal medium. After 24 h the supernatants were collected and filtered through a 100 kDa membrane to remove the virus. The ultrafiltrated supernatants, 0.3 ng/mL TGFβ1 or the combination were then used to stimulate HBF cells. After 48 h, western blotting was used to quantify protein levels of myofibroblast markers such as α-smooth muscle actin (α-SMA). Further analyses are planned using RT-PCR, immunocytochemistry and transmission electron microscopy to identify phenotypic myofibroblast-type cells.

**Results:** HBF cells stimulated with ultrafiltrated HBE-infected supernatants and TGFβ1 in combination demonstrated significantly increased α-SMA protein on western blotting compared to supernatants from non-infected HBE cells combined with TGFβ1, or TGFβ1 alone. HRV-infected epithelial cell supernatants alone did not induce α-SMA protein production.

**Conclusions:** Human rhinovirus and TGFβ1 may have a synergistic role in inducing fibroblast-to-myofibroblast differentiation. Understanding the molecular mechanisms of this synergy may identify therapeutic approaches to attenuate the remodeling process, which is characteristic of asthma in early life.

### A18 Interleukin-4 and interleukin-13 protein and gene expression from birch allergen stimulated peripheral blood mononuclear cells before and after nasal allergen challenge

#### Matthew Rawls^1,2^, Mark W. Tenn^1,2^, Jenny Thiele^1,2^, Lisa M. Steacy^2^, Daniel E. Adams^2^, Anne K. Ellis^1,2^

##### ^1^Departments of Medicine and Biomedical & Molecular Science, Queen’s University, Kingston, Ontario, Canada; ^2^Allergy Research Unit, Kingston General Hospital, Kingston, Ontario, Canada

###### **Correspondence:** Matthew Rawls

*Allergy, Asthma & Clinical Immunology* 2018, **14(Suppl 1):**A18

**Background:** Allergic rhinitis (AR) is currently recognized as a T helper cell type 2 (Th2) driven disease with typically increased levels of relevant cytokines, such as interleukin (IL)-4 and IL-13 found locally in nasal tissues. The aim of the current study was to determine if nasal allergen challenge (NAC) could affect IL-4 and IL-13 protein and gene expression in birch-stimulated peripheral blood mononuclear cells (PBMCs) cultures.

**Methods:** Consenting individuals (4 birch-allergic and 5 non-allergic) were administered increasing concentrations of birch pollen extract intranasally until each participant achieved qualifying criteria for a positive NAC [Total Nasal Symptom Score (TNSS) ≥ 8 and % Peak Nasal Inspiratory Flow (PNIF) fall ≥ 50%]. At baseline and 4 h post-NAC, peripheral blood samples were collected, PBMCs isolated, and stimulated for 5 days with birch pollen extract or extract diluent. Cytokines were measured using the Bio-Plex^®^ 200 system and mRNA expression was assessed through qPCR. Statistical analysis was performed using GraphPad Prism 7.0.

**Results:** Baseline PBMCs post-NAC expressed significantly decreased IL-4 levels (p = 0.0313) and birch-stimulated PBMCs post-NAC demonstrated a decreasing trend for IL-4 levels (p = 0.0742) compared to pre-NAC. No significant difference was observed for PBMC derived IL-13 levels before and after the NAC. There was no significant difference between allergic and non-allergic PBMCs. Trends were observed for increased IL-4 mRNA expression in allergic PBMCs compared to non-allergic PBMCs pre-NAC (p = 0.1111), increased IL-13 mRNA expression in non-allergic birch stimulated PBMCs post-NAC compared to pre-NAC (p = 0.0625), and decreased IL-13 levels post-NAC from non-allergic PBMCs in the diluent condition (p = 0.0625).

**Conclusions:** An expected increase of IL-4 and IL-13 protein and mRNA expression in allergic participants was not observed. This unpredicted result may be a consequence of a low sample size or cellular fatigue after repeated exposure to birch pollen extract.

### A19 Efficacy and safety of intranasal corticosteroids approved for over-the-counter use

#### Geoffrey Saroea

##### Department of Medical Affairs, GlaxoSmithKline Consumer Health, Mississauga, Ontario, Canada

###### **Correspondence:** Geoffrey Saroea

*Allergy, Asthma & Clinical Immunology* 2018, **14(Suppl 1):**A19

**Background:** Allergic rhinitis (AR) is a common disorder affecting 20–25% of Canadians. Intranasal corticosteroids (INCS) are a mainstay of AR treatment, and some are available over-the-counter (OTC). The aim of this review was to evaluate the efficacy and safety of intranasal corticosteroids approved for OTC use.

**Methods:** A search using keywords allergic rhinitis, anti-allergic agents, intranasal administration, fluticasone, and triamcinolone was conducted on Ovid MEDLINE and Google Scholar. The search was limited to placebo-controlled studies published from 1991 to present. Studies were included which evaluated the efficacy of intranasal fluticasone propionate or triamcinolone acetonide, for the treatment of seasonal or perennial allergic rhinitis.

**Results:** Six trials met the inclusion criteria, three evaluating fluticasone propionate intranasal spray (FPNS), and three evaluating triamcinolone acetonide intranasal spray (TANS), versus placebo. A total of 1218 and 747 subjects were enrolled in FPNS and TANS trials, respectively. The primary efficacy measure was reduction in nasal symptom scores (NS). FPNS demonstrated statistically significant reduction in nasal symptoms when compared to placebo (p < 0.01) and reduction in obstruction upon awakening (p < 0.01), indicating efficacy lasting 24 h. One study evaluating reduction in ocular symptoms (OS) showed FPNS significantly reduced OS when compared with placebo (p = 0.002). TANS also demonstrated significant reduction in NS when compared to placebo (p < 0.05) across all trials. Overall, safety evaluations indicated both FPNS and TANS were well tolerated.

**Conclusions:** As more INCSs for AR become available OTC, the role of the health professional is pivotal in diagnosis, treatment selection, and education of patients with AR. The efficacy and safety of INCS is well established. ARIA and Canadian Guidelines recommend the use of INCS to manage mild persistent to moderate-severe allergic rhinitis. Health professionals should feel comfortable in recommending an INCS for use in these patients.

### A24 Longitudinal analysis of stability of immune and physiological biomarkers of asthma

#### Nami Shrestha Palikhe^1^, Ana-Maria Bosonea^1^, Cheryl Laratta^1^, Vivek Dipak Gandhi^1^, Drew Nahirney^1^, Angela Hillaby, Miranda Bowen, Mohit Bhutani^1,2^, Irvin Mayers^1^, Lisa Cameron^1,3^, Harissios Vliagoftis^1,2^

##### ^1^Division of Pulmonary Medicine, Department of Medicine, University of Alberta, Edmonton, Alberta, Canada; ^2^Alberta Asthma Center, University of Alberta, Edmonton, Alberta, Canada; ^3^Department of Pathology, Schulich School of Medicine & Dentistry, Western University, London, ON, Canada

###### **Correspondence:** Nami Shrestha Palikhe

*Allergy, Asthma & Clinical Immunology* 2018, **14(Suppl 1):**A24

**Background:** We reported two potential biomarkers for asthma severity, namely circulating CD4^+^ CRTh2^+^ T-cells and CD14^++^CD16^+^ PAR2^+^. Chemoattractant receptor-homologous molecule expressed on Th2 cells (CRTh2) is a receptor for PGD2 and expressed by T cells, eosinophils, basophils and type 2 innate lymphoid cells (ILC2). Protease-Activated Receptor-2 (PAR-2) is a pro-inflammatory receptor activated by serine proteases and expressed on many cells, including monocytes and lung resident cells. Our next step to validate the utility of flowcytometric measures as biomarkers for asthma severity was to assess the stability of these markers in asthmatics over time.

**Methods:** Stability of “% of CD4^+^ CRTh2^+^T cells” and “% of CD14^++^CD16^+^PAR-2^+^ monocytes” in peripheral blood was studied in asthmatics (n = 19) by flowcytometry. FEV_1_ and asthma control questionnaire based on 7 (ACQ7) and 5-point scale (ACQ5) as physiological variables were collected. The stability of total numbers of ILC2 and eosinophils were analysed. Within person stability of laboratory values over 4 visits were calculated using the intraclass correlation (ICC) by R version 3.4.0.

**Results:** The mean age of asthmatics in our study was 45 years. The stability of % of CD4^+^CRTh2^+^ T-cells (ICC = 0.20) and % of CD14^++^CD16^+^PAR-2^+^ (ICC = 0.24) was poor over four visits. Analysis of ICC suggested high stability over the four repeated values in FEV_1_ (ICC = 0.90), ACQ5 (ICC = 0.68) and ACQ7 (ICC = 0.75). ICCs were moderate for % of eosinophils (ICC = 0.44) and ILC2 (ICC = 0.45). No correlations were observed between immune cell profiles, FEV_1_, ACQ5 and ACQ7.

**Conclusions:** % CD4^+^CRTh2^+^ T-cells and % CD14^++^CD16^+^ PAR-2^+^ cells varied substantially over the four visits in this asthmatic population. Variability did not correlate with physiological measures of asthma or questionnaire measures of asthma control. The reason for this variability is not known. This study also showed good stability of FEV_1_, ACQ5 and ACQ7 and moderate stability of % of ILC2 and % of eosinophils.

### A26 Observed reduction of healthcare utilization after Omalizumab initiation among patients with persistent asthma followed in clinical settings in Ontario, Canada

#### William H. Yang^1^, Remi Gagnon^2^, Eliofotisti Psaradellis^3^, Emmanouil Rampakakis^3^, Jean-Louis Stril^4^, Sara Chehab^4^

##### ^1^Ottawa Allergy Research Corporation, University of Ottawa Medical School, Ottawa, Ontario, Canada; ^2^Centre Hospitalier de L’Université Laval (CHUL), Quebec City, Quebec, Canada; ^3^JSS Medical Research, St-Laurent, Quebec, Canada; ^4^Novartis Pharmaceuticals Canada Inc., Dorval, Quebec, Canada

###### **Correspondence:** Jean-Louis Stril

*Allergy, Asthma & Clinical Immunology* 2018, **14(Suppl 1):**A26

**Background:** In Canada, it is estimated that asthma affects 8.5% of the total population. It is the leading cause of hospital admissions, the third leading cause of work loss, and results in 146,000 emergency room visits annually in the overall population. Omalizumab is indicated for the treatment of adults and adolescents with moderate to severe persistent allergic asthma whose symptoms are inadequately controlled despite optimized standard therapy. Real world effectiveness data assessing the HCU in the Canadian context is limited.

**Objective:** This study was a retrospective, pre-post cohort, observational study. The primary objective was to evaluate the health care utilization (HCU) following Omalizumab initiation as assessed by the reduction in number of hospitalizations, emergency room (ER) visits, and oral corticosteroid (OCS) use in patients covered in Ontario. The number of night awakenings was an exploratory endpoint.

**Results:** 148 patients (mean age 57.6; female 62.2%) formed the study population. Omalizumab was associated with a 74.4% reduction in the number of hospitalization (pre vs post-Omalizumab’s 12 month treatment period: 0.7 vs 0.2 p < 0.001). 89.9% of patients did not have any asthma related hospitalization. There was a reduction of 87.5% in ER visits (7.3 vs. 0.9 p < 0.001), 66.2% of patients did not have any emergency visit. A 74.7% reduction of the number of high dose OCS by (4.23 vs. 1.07 p < 0.001), 52.7% of patients did not need to take any courses of high dose OCS. The mean number of night awakenings/per week decreased from 6.1 (8.03) to 1.3 (2.79) following 12 month treatment with Omalizumab.

**Conclusions:** There was an observed reduction in the number of hospitalizations, ER visits, and high-dose OCS courses post-Omalizumab use in patients with severe uncontrolled asthma in a real-world setting. The results are consistent with outcomes observed in previous large real-world trials such as the experience registry.

### A27 Comparative analysis of total ocular and total rhinoconjunctivitis symptom profiles in the Environmental Exposure Unit versus the Nasal Allergen Challenge model

#### Mark W. Tenn^1,2^, Lisa M. Steacy^2^, Jenny Thiele^1,2^, Daniel E. Adams^2^, Terry J. Walker^2^, Anne K. Ellis^1,2^

##### ^1^Departments of Medicine and Biomedical & Molecular Sciences, Queen’s University, Kingston, Ontario, Canada; ^2^Allergy Research Unit, Kingston General Hospital, Kingston, Ontario, Canada

###### **Correspondence:** Mark W. Tenn

*Allergy, Asthma & Clinical Immunology* 2018, **14(Suppl 1):**A27

**Background:** The Environmental Exposure Unit (EEU) and Nasal Allergen Challenge (NAC) are both experimental models of Allergic rhinitis (AR). They mimic the inflammatory processes and symptom manifestations associated with exposure to sensitized aeroallergens. Previous studies demonstrated a unique Total Nasal Symptom Score (TNSS) profile following allergen challenge in each model. As AR individuals also experience ocular symptoms, we sought to compare the Total Ocular Symptom Score (TOSS) profiles following allergen challenge in both the EEU and NAC models.

**Methods:** 7 birch-allergic and 4 non-allergic individuals who participated in both an EEU study and a NAC study using birch pollen were included in the analysis. For both studies, TNSS, TOSS, and Total Rhinoconjunctivitis Symptom Scores (TRSS) were collected at baseline, 15 min (NAC only), 30 min, and hourly until 12 h post-challenge (every half hour during first 4 h for EEU). Data was analyzed using GraphPad Prism. Adjustments for multiplicity testing were not made.

**Results:** Peak increases in TOSS were observed at 3 h and 30 min post-challenge in the EEU and NAC respectively. However, they were only significant in the EEU (p < 0.05). In contrast, TRSS peaked at 3 h (p < 0.05) and 15 min (p < 0.05) post-challenge in the EEU and NAC respectively. Overall, a significantly higher mean TRSS (p < 0.05), but not TOSS (p = 0.16), was observed in the EEU compared to the NAC. When comparing TOSS profiles from both models by time point, significant differences were observed at 30 min, 1, 3, 4, 5, 7, and 9 h (all p < 0.05). Conversely, significant differences in TRSS profiles were observed at 30 min, 1 through 9 and 12 h (all p < 0.05) following allergen challenge.

**Conclusions:** Significant ocular symptoms in the EEU may be attributable to the prolonged pollen exposure period and contact with airborne pollen. Significant differences in TRSS within and between each model were primarily driven by differences in TNSS, not TOSS.

### A29 Predicting the atopic March: results from the Canadian Healthy Infant Longitudinal Development (CHILD) Study

#### Maxwell M. Tran^1^, Diana L. Lefebvre^1^, Christoffer Dharma^1^, David Dai^1^, Wendy Y.W. Lou^2^, Padmaja Subbarao^3^, Allan B. Becker^4^, Piush J. Mandhane^5^, Stuart E. Turvey^6^, Malcolm R. Sears^1^

##### ^1^Department of Medicine, McMaster University, Hamilton, Ontario, Canada; ^2^Dalla Lana School of Public Health, University of Toronto, Toronto, Ontario, Canada; ^3^Department of Pediatrics, University of Toronto & Hospital for Sick Children, Toronto, Ontario, Canada; ^4^Department of Pediatrics & Child Health, University of Manitoba, Winnipeg, Ontario, Canada; ^5^Department of Pediatrics, University of Alberta, Edmonton, Ontario, Canada; ^6^Department of Pediatrics, University of British Columbia, Vancouver, Ontario, Canada

###### **Correspondence:** Maxwell M. Tran

*Allergy, Asthma & Clinical Immunology* 2018, **14(Suppl 1):**A29

**Background:** The ‘atopic March’ describes the progression from atopic dermatitis during infancy to asthma and allergic rhinitis in later childhood. In a Canadian birth cohort, we investigated whether concomitant allergic sensitization enhances subsequent development of these allergic diseases at age 3 years.

**Methods:** At age 1, children completed skin prick testing. Children were considered sensitized if they produced a wheal 2 mm or greater than the negative control to any of ten inhalant or food allergens. Children were also assessed for atopic dermatitis using the diagnostic criteria of the UK Working Party. At age 3, children were assessed for asthma, allergic rhinitis, food allergy, and atopic dermatitis. Data from 2311 children were available.

**Results:** Atopic dermatitis without allergic sensitization was not associated with an increased risk of asthma at age 3, after adjusting for common confounders (RR 0.46, 95% CI 0.11–1.93). Conversely, atopic dermatitis with allergic sensitization increased the risk of asthma over sevenfold (RR 7.04, 95% CI 4.13–11.99). Atopic dermatitis and allergic sensitization had significant interactions on both the additive (RERI 5.06, 95% CI 1.33–11.04) and multiplicative (ratio of RRs, 5.80, 95% CI 1.20–27.83) scales in association with asthma risk. There was also a positive additive interaction between atopic dermatitis and allergic sensitization in their effects on food allergy risk (RERI 15.11, 95% CI 4.19–35.36).

**Conclusions:** Atopic dermatitis without allergic sensitization was not associated with an increased risk of asthma. In combination, atopic dermatitis and allergic sensitization had strong interactive effects on both asthma and food allergy risk at age 3.

### A30 An update - the observed incidence of adverse reactions in patients receiving Omalizumab therapy in a tertiary allergy and asthma clinic in Canada

#### Jodi Valois^1^, Jenna Falbo^1^, Stephanie Santucci^1^, Jacob Karsh^2^, William H. Yang^1,2^

##### ^1^Ottawa Allergy and Research Centre, Ottawa, Ontario, Canada; ^2^University of Ottawa Medical School, Ottawa, Ontario, Canada

###### **Correspondence:** Jodi Valois

*Allergy, Asthma & Clinical Immunology* 2018, **14(Suppl 1):**A30

**Background:** In a post-marketing analysis last updated in July 2007, the FDA reported that an estimated 0.2% of patients suffered treatment related anaphylaxis and rare incidence of serum sickness. To substantiate this, the occurrence of treatment related anaphylaxis and serum sickness was assessed in one of the largest global allergy and asthma tertiary clinics.

**Methods:** The original retrospective chart review of our database of Omalizumab administration was performed to collect data between 1998 and June 2014. This has been updated to include the recent data from July 2014 to June 2017. Data was collected on the number of patients receiving treatment, the number of injections received/dose schedule of each patient, as well as the age and gender of each patient.

**Results:** During clinical trials and with our post market experience, between 1998 and June 2017, over 50,000 injections of Omalizumab were administered to over 400 patients diagnosed with either severe allergic asthma or chronic idiopathic urticaria. No cases of anaphylaxis or serum sickness like symptoms were observed.

**Conclusions:** Meticulous care was taken by our Omalizumab administration clinic to ensure optimal safety based on the emphasized warnings of anaphylaxis, as well as the indicated warnings and precautions for serum sickness. Data collected in this analysis observed no cases of anaphylaxis or serum sickness like symptoms in the treatment of patients with Omalizumab, during a period of 17.5 years, thus confirming the low incidence of both anaphylaxis and serum sickness.

## Food Allergy/Anaphylaxis

### A32 Food allergy knowledge translation gaps among a national cohort of pediatric allergists (FAKT-gaps)

#### Elissa M. Abrams^1^, Lianne Soller^2^, Matthew Greenhawt^3^, David M. Fleischer^4^, Alexander G. Singer^5^, Edmond S. Chan^6^

##### ^1^Department of Pediatrics, Section of Allergy and Clinical Immunology, University of Manitoba, Winnipeg, Manitoba, Canada; ^2^Post Doctoral Fellow, University of British Columbia, Vancouver, British Columbia, Canada; ^3^Assistant Professor of Pediatrics, Section of Allergy, University of Colorado Denver School of Medicine, Denver, Colorado, United States of America; ^4^Associate Professor of Pediatrics, Section of Allergy, University of Colorado Denver School of Medicine, Denver, Colorado, United States of America; ^5^Assistant Professor, Department of Family Medicine, University of Manitoba, Winnipeg, Manitoba, Canada; ^6^Associate Professor, Division of Allergy and Immunology, Department of Pediatrics, University of British Columbia, Vancouver, British Columbia, Canada

###### **Correspondence:** Elissa M. Abrams

*Allergy, Asthma & Clinical Immunology* 2018, **14(Suppl 1):**A32

**Background:** The approach to peanut allergy prevention has shifted with publication of the Learning Early About Peanut (LEAP) trial and recently released NIAID guideline.

**Objective:** To determine allergists’ interpretation of LEAP findings, and assess feasibility of NIAID guideline implementation.

**Methods:** A 29-question survey was distributed to Canadian allergists through the Canadian Society of Allergy and Clinical Immunology.

**Results:** 64.4% (95% CI 52.3–75.3) of allergists were pediatric trained. There was variability in how allergists define infants at high-risk of peanut allergy. Only 37.7% (95% CI 26.3–50.2) of allergists routinely perform infant oral food challenges (OFCs). Pediatric allergists were 6.67 (95% CI 10.9–31.3) times more likely to perform OFCs than internal medicine trained allergists. When performed for high-risk infants with a positive skin prick test (SPT), only 19.7% (95% CI 10.9–31.3) of allergists would do a same day OFC.

**Conclusions:** The definition of high-risk requires international consensus, as allergists may SPT infants not classified as high-risk by the NIAID guideline, increasing the potential for false positive SPTs before ingestion. Few allergists perform infant OFCs routinely (especially with intermediately positive SPTs), and few are done on the same day as positive SPTs, potentially resulting in peanut avoidance indefinitely in many infants.

### A33 Usability and comprehension of an illustrated Canadian anaphylaxis action plan for kids (Kids’ CAP study)

#### Waleed Alqurashi^1,2^, Alisha Awadia^2^, Annie Pouliot^3^, Michel Cloutier^3^, Simon Hotte^1^, Lauren Segal^1^, Danica Irwin^3^, Régis Vaillancourt^2,3^

##### ^1^Department of Pediatrics, University of Ottawa, Ottawa, Ontario, Canada; ^2^Clinical Research Unit, Children’s Hospital of Eastern Ontario, Ottawa, Ontario, Canada; ^3^Pharmacy Department, Children’s Hospital of Eastern Ontario, Ottawa, Ontario, Canada

###### **Correspondence:** Alisha Awadia

*Allergy, Asthma & Clinical Immunology* 2018, **14(Suppl 1):**A33

**Background:** We designed a written anaphylaxis plan called Canadian Anaphylaxis Action Plan for Kids (Kids’ CAP) which incorporates validated pictograms with written instructions. Using a patient-centered approach, we aimed to assess the impact of the Kids’ CAP on anaphylaxis recognition and treatment, and to determine its perceived usefulness.

**Methods:** Prior to clinical validation, we assessed the readability and understandability metrics of the Kids’ CAP using the Gunning Fog index (GFI), the Fry index, and the Patient Education Material Assessment Tool (PEMAT). During the clinical assessment phase, patients (12–17 years of age) and parents of patients (0–11 years of age) were taught and given the Kids’ CAP during first visit with an allergist at two community clinics, or an anaphylaxis emergency department (ED) visit at a tertiary pediatric centre. The Newest Vital Sign tool was used to determine the baseline health literacy level of study participants. Three weeks later, we conducted a phone interview to assess their comprehension of anaphylaxis manifestations and management. We also used the Consumer Information Rating Form (CIRF) to measure the participants’ perception of the usability, satisfaction, and usefulness of the Kids’ CAP.

**Results:** Two hundred participants completed the follow-up interview. The median age of patients was 3.95 years (IQR 1.4–9.2), and 104/200 (52%) were males. The written contents of the Kid’s CAP matched grade 7 readability level. The mean CIRF score for usability and design quality were 23 (out of 25) (SD 2.6), and 25.1 (out of 30) (SD 2.5), respectively. The mean comprehension score was 11.2 (out of 14) (SD 1.7) with no significant difference between participants with and without previous experience with anaphylaxis, or high versus low literacy level.

**Conclusions:** The implementation of the Kids’ CAP can improve patient’s comprehension of anaphylaxis manifestations and treatment, which are critical components of ED management of anaphylaxis.

### A34 Structured environment may protect primary school aged children from food allergic reactions

#### Jeremy H. Biro^1^, Julia Upton^2,3^, Eyal Grunebaum^1,2,3^

##### ^1^Developmental & Stem Cell Biology, Hospital for Sick Children, Toronto, Ontario, Canada; ^2^Department of Pediatrics, University of Toronto, Toronto, Ontario, Canada; ^3^Clinical Immunology and Allergy, Hospital For Sick Children, Toronto, Ontario, Canada

###### **Correspondence:** Jeremy H. Biro

*Allergy, Asthma & Clinical Immunology* 2018, **14(Suppl 1):**A34

**Background:** The recent rise in food allergy prevalence has led to an increase in the number of parents concerned about accidental exposure of their children to allergenic food. Our goal was to investigate the role of the school environment on food allergy emergency room visits.

**Methods:** Visits to Canadian emergency departments for food and environmental/drug allergic and anaphylactic reactions were obtained from the Canadian Institute for Health Information (CIHI), based on the 2013–2014 National Ambulatory Care Reporting System. Reactions were defined in accordance to the ICD-10-CA diagnostic codes. Data was provided grouped by approximate school attendance ages into 3–5 (pre-school), 6–12 (primary school) and 13–18 (secondary school) years. Data was divided into day (9–16:59) and night (17–0:59). Allergic reactions to Environmental and Drug triggers were analyzed as control. p < 0.05 was considered as statistically significant assuming equal distribution. Chi squared analysis and T-testing (STATA14) were performed. Numbers shown are normalized by age categories and represent mean per hour over the span of the 6 months with full school scheduling.

**Results:** During the study period, 2758 food and 3398 environmental/drug visits were recorded. Significantly fewer food allergic reactions were recorded during the day than at night for preschool (5.17 ± 2.16 vs. 9.83 ± 4.46), elementary (2.875 ± 1.13 vs. 5.98 ± 2.53), and secondary school (4.65 ± 1.89 vs. 7.06 ± 2.35) age groups. Fewer events were recorded among primary school ages during the day than both preschool and secondary ages, while this difference was not significant at night. Environmental/drug allergic reactions showed the opposite trend, with fewer reactions at night during the day, and no differences between age groups during day or night.

**Conclusions:** A structured environment may protect primary school aged children from food allergic reactions during daytime. In general, school-aged children visit the emergency room more frequently after school, which could inform recruitment for allergy studies.

### A35 Anaphylaxis incidence and definitions from population-based research: a systematic review

#### Derek K. Chu, Susan Waserman

##### Division of Clinical Immunology & Allergy, Department of Medicine, McMaster University, Hamilton, Ontario, Canada

###### **Correspondence:** Derek K. Chu

*Allergy, Asthma & Clinical Immunology* 2018, **14(Suppl 1):**A35

**Background:** The risk of developing anaphylaxis has been reported in multiple population-based studies, with estimates and definitions being highly variable. The aim of this systematically review is to synthesize the incidence, etiologies, and definitions of anaphylaxis worldwide.

**Methods:** Following established guidelines, systematic review of records in any language obtained from multiple database searches (MEDLINE, EMBASE, Cochrane Register) through May 25, 2017 for population-based studies reporting on the incidence and elicitors of anaphylaxis and methodology used to identify and validate cases. Risk of bias and assessment of the certainty of evidence will be rated by the Grading of Recommendations, Assessment, Development and Evaluation (GRADE) approach.

**Results:** Database searches yielded 1706 unique records, of which 245 were potentially relevant, and 91 are included for analysis. The range of reported incidence rates for anaphylaxis, stratified by geographic location, age, setting and definition will be reported. Methods of identification and validation of anaphylaxis will be reviewed, highlighting their relative merits and potential limitations. We anticipate that like most population-based research, the main limitation of these studies will be under ascertainment of events. If there is sufficient detail in reporting, we will synthesize the incidence and accuracy of specific causes of anaphylaxis, such as foods, drugs, insects, and idiopathic.

**Conclusions:** The incidence of anaphylaxis through population-based research will be systematically reviewed, synthesized and assessed. These data, and a uniform case definition are critical to establish anaphylaxis risk estimates for patients and clinicians, as well as to inform future research and guidelines.

### A36 Novel suspension medium for peanut and tree nut oral immunotherapy – Canadian pediatric private practice oral immunotherapy using NuT suspension (Canadian - PPPOINTS)

#### Douglas P. Mack^1,2,^ Mariam A. Hanna^1,2^

##### ^1^Halton Pediatric Allergy, Burlington, Ontario, Canada; ^2^Department of Pediatrics, McMaster University, Hamilton, Ontario, Canada

###### **Correspondence:** Douglas P. Mack

*Allergy, Asthma & Clinical Immunology* 2018, **14(Suppl 1):**A36

**Background:** As the incidence of food allergies continues to rise, potential therapies are undergoing investigation. Oral immunotherapy (OIT) for food has been studied in the pediatric population in both phase 2 and ongoing phase 3 trials. Families are keenly interested in this approach of actively managing food allergy despite our lack of knowledge of long term outcomes. We sought to investigate the use of a novel suspension medium to desensitize patients with peanut and tree nut allergy.

**Methods:** This is a retrospective review from 2014 to 2017 of OIT in a pediatric outpatient clinic. After initial evaluation, testing, detailed informed consent/assent and potential oral challenge, participants were given the opportunity to be desensitized to the foods of concern. This included peanuts, and multiple simultaneous tree nuts. Peanut and/or individual tree nut flours were suspended in Suspendit™ medium and were given daily as sublingual/oral administration starting at 0.25 mg total nut protein and progressing up to 50 mg nut protein in suspension and then transitioned to actual weighed nut. Doses were increased in clinic on a biweekly basis.

**Results:** Oral desensitization was successfully completed in a high majority of the 38 patients who initially enrolled ages 9 months to 17 years. The majority of individuals reported mild side effects. However, moderate to severe reactions were reported in several individuals during build up and maintenance requiring epinephrine use. Cofactors for reactions such as illness and compliance were identified. Side effects diminished with prolonged use. Skin prick testing decreased after being on maintenance therapy for 6–12 months.

**Conclusions:** This is the first Canadian data to report on the safety and efficacy of pediatric oral immunotherapy in private practice for peanut and tree nut allergies. Suspendit™ suspension medium allowed for safe and effective dosing titration of OIT for peanuts and tree nuts.

### A37 Safety profile of the first Canadian randomized controlled trial of oral immunotherapy with cow’s milk in children

#### Sarah De Schryver^1^, Bruce Mazer^1^, Ann Clarke^2^, Yvan St. Pierre^3^, Duncan Leytneyi^1^, Alexandra Langlois^1^, Bahar Torabi^1^, Edmond Chan^4^, Moshe Ben-Shoshan^1^

##### ^1^Division of Allergy and Immunology, Department of Pediatrics, McGill University, Montreal, Quebec, Canada; ^2^Division of Rheumatology, Department of Medicine, University of Calgary, Calgary, Alberta, Canada; ^3^Division of Clinical Epidemiology, Department of Medicine, McGill University Health Center, Montreal, Quebec, Canada; ^4^Division of Allergy and Immunology, Department of Pediatrics, University of British Columbia, Vancouver, British Columbia, Canada

###### **Correspondence:** Sarah De Schryver

*Allergy, Asthma & Clinical Immunology* 2018, **14(Suppl 1):**A37

**Background:** Cow’s milk oral immunotherapy (OIT) is effective in attaining desensitization but its safety is not well established. We aimed to characterize adverse events (AE) in a pediatric cohort undergoing OIT at the Montreal Children’s Hospital.

**Methods:** Data was collected on AE occurring during OIT between 2013–2017. Anaphylaxis was defined as involvement of 2 organ systems and/or hypotension in response to a milk exposure. Non-anaphylactic AE was defined when only one organ system was affected as previously published. Descriptive statistics were used to represent demographics, clinical characteristics and co-morbidities. Poisson regression was performed to evaluate risk factors associated with anaphylaxis.

**Results:** Among 29 children, the mean age at study entry was 11.9 years (SD 3.9) and the majority were male (58.6%). Co-morbidities included asthma (82.8%), seasonal allergy (48.3%) and eczema (27.6%).In total 8 children withdrew from OIT (27.6%). Among those, the mean number of anaphylactic reactions per patient was 14.1 (SD 18.2) versus 7.2 (SD 7.3) in non-withdrawals. On average, 0.6 (SD 0.9) reactions required epinephrine treatment in children maintained in the study versus 0.7 (SD 1.1) in withdrawals. Differences between both groups were statistically non-significant.Among 1060 AE, the majority were non-anaphylactic (75%). Of those, the majority occurred during the escalation phase (91.0%) and were categorized as mild (78.5%). Risk factors for anaphylaxis included older children at study entry [risk ratio: 1.09 (95% CI 1.04, 1.14)], active eczema [3.67 (95% CI 2.65, 5.09)] and greater baseline skin prick test size [1.09 (95% CI 1.05, 1.14)]. Boys and children with well-controlled asthma, were less likely to develop anaphylaxis [0.71 (95% CI 0.52, 0.95)] and [0.39 (95% CI 0.24, 0.63)] respectively.

**Conclusions:** AE occur frequently during OIT. Caregivers should be vigilant regarding this risk, especially in older children, with active eczema and larger baseline skin test size.

### A38 Barriers to oral food challenge (OFC) implementation in Canada

#### Elaine Hsu^1^, Christopher Mill^1^, Lianne Soller^1^, Elissa Abrams^2^, Edmond S. Chan^1^

##### ^1^Division of Allergy and Immunology, Department of Pediatrics, Faculty of Medicine, University of British Columbia, BC Children’s Hospital, Vancouver, British Columbia, Canada; ^2^Pediatric Allergy and Clinical Immunology, University of Manitoba, Winnipeg, Manitoba, Canada

###### **Correspondence:** Elaine Hsu

*Allergy, Asthma & Clinical Immunology* 2018, **14(Suppl 1):**A38

**Background:** Oral food challenges (OFCs) are considered the gold standard in food allergy diagnostic testing. Despite this, there is a lack of Canadian data regarding its implementation. We investigated how many allergists perform OFCs, number of OFCs performed, barriers that prevent more from being conducted, and possible solutions to these barriers.

**Methods:** Surveys were sent to allergist members of the Canadian Society of Allergy and Clinical Immunology (CSACI). Questions included practice characteristics, number of OFCs conducted, barriers to conducting more OFCs, and solutions that would mitigate barriers.

**Results:** Of the 205 CSACI allergists surveyed, 61 responded (response rate = 30%). Preliminary data showed 50 of the 61 respondents conducted OFCs (range = 1–60/month, median = 12/month, 66% perform 10 or more/month). There was no significant difference between number of OFCs performed and allergists’ years of practice, or number of OFCs performed between allergists using a specific hospital OFC fee code and those using all other codes. Nearly all respondents agreed there was a need to conduct more OFCs, but barriers exist, with 74% of respondents choosing “lack of support staff”, and 70% choosing “lack of time” as “moderately to extremely influential” barriers. The most influential solutions were “standard criteria for which challenges should be done in a hospital versus the community” chosen by 72% of respondents, and “patients agreeing to consume the food after a negative OFC”, chosen by 70%.

**Conclusions:** This is the first study to capture barriers and examine possible solutions to OFC implementation. Selection bias may be a limitation in this study since allergists who perform OFCs may be more likely to complete the survey. Results show that resources (support staff and time) to perform OFCs are lacking, and that standard OFC protocols and patient education around OFCs need to be explored as possible solutions to allow OFCs to be more accessible to Canadian patients.

### A39 The lived experience of teens with food-induced anaphylaxis (FIA): a proposed study

#### Sara F. Johnson, Roberta L. Woodgate

##### College of Nursing, Rady Faculty of Health Sciences, University of Manitoba, Winnipeg, Manitoba, Canada

###### **Correspondence:** Sara F. Johnson

*Allergy, Asthma & Clinical Immunology* 2018, **14(Suppl 1):**A39

**Background:** Teens with food induced anaphylaxis (FIA) live with the unpredictable risk of reacting to, and potentially dying from something they eat. FIA results in considerable burden for teens, including stigma, marginalization, and the need for constant vigilance. Despite teen anaphylaxis rates in Canadian emergency rooms more than doubling in recent years, no published studies have focused exclusively on the teen experience living with FIA in Canada. At present, we lack the knowledge to recommend best practices in managing teens with FIA. Therefore, in this study we aim to create a detailed understanding of teens’ lived experience of FIA in daily life.

**Methods:** We combine van Manen’s hermeneutic phenomenology with photovoice techniques to explore and interpret the lives of teens with FIA through their eyes, using their own words. Participants will include teens (age 12–19) living in Manitoba. Each teen will participate in 2 semi-structured interviews: the first will focus on lived experience, with photovoice images and techniques incorporated in the second interview. Interviews will be recorded and transcribed, and images retained for analysis. Both researchers will code and analyze data independently to identify meaning, relationships, and themes. Data collection and analysis will occur concurrently, informing the study as it progresses. Teens will be invited to join a Youth Advisory Committee (YAC) to discuss findings and inform knowledge translation efforts.

**Results:** To be presented via traditional academic means (e.g. journal publications, conference presentations) and tailored education sessions for knowledge users, alongside initiatives determined in consultation with YAC members.

**Conclusions:** This study fosters understanding, forming a base for future research to meet the healthcare needs of these teens and their families. Findings have potential to inform policy affecting teens with FIA, helping to focus change in priority areas and assist in health promotion for teens and families living with FIA.

### A40 Canadian survey of patient and caregiver knowledge of food allergy

#### Ling Ling^1^, Lana Rosenfield^1^, Ernie Avilla^2^, Laurie Harada^3^, Marilyn Allen^3^, Susan Waserman^1^

##### ^1^Division of Clinical Immunology and Allergy, McMaster University, Hamilton, Ontario, Canada; ^2^McMaster University, Hamilton, Ontario, Canada; ^3^Food Allergy Canada, Toronto, Ontario, Canada

###### **Correspondence:** Ling Ling

*Allergy, Asthma & Clinical Immunology* 2018, **14(Suppl 1):**A40

**Background:** Patient and caregiver knowledge of the symptoms and triggers of food allergy is key to successful management. To date, there have been no Canadian studies on this issue. We aimed to characterize patient and caregiver knowledge of food allergy in a Canadian setting.

**Methods:** The Food Allergy Epinephrine Auto-Injector Study (FEAST) is an observational study on the dining experience of food allergic patients. Online surveys were administered to patients and caregivers in February 2015. Patients were identified from Food Allergy Canada’s database. Among others, the survey contained questions on symptoms and triggers of food allergy.

**Results:** There were 1165 respondents of the survey. 93.3% correctly identified the symptoms of an allergic reaction. Most knew that ingestion of small amounts of allergen could cause a reaction (92.6%). There was a high degree of understanding that removal of allergen from a plate is not sufficient to prevent reaction (93%). More respondents (48.8%) than not (45.5%) believed smell of peanut butter could cause a reaction. The majority correctly identified Health Canada’s list of the ten most common food allergens: peanut (93.7%), treenuts (93.2%), shellfish and crustaceans (92.3%), egg (91.5%), milk (90.4%), wheat (84.1%), soy (81.2%), fish (77.3%), sesame (76.1%), mustard (57.3%), sulfites (not tested). There was a high degree of correct identification of uncommon causes of food allergy: apples (97.3%), chocolate (93.6%) coconut (94%), strawberry (77.4%), onion (98.6%), garlic (98.3%), pumpkin seed (98%).

**Conclusions:** The Canadian respondents had high recognition of the symptoms of food allergy. Knowledge of the common and uncommon food allergens was also good. Understanding of cross contamination as a cause of allergic reaction was similarly high. Lower rate of correct identification of mustard and strawberry as common and uncommon allergens respectively were seen. There was also a notable amount of misunderstanding that smell could cause a reaction.

### A41 The use of oral immunotherapy for milk allergy in a Canadian pediatric private practice clinic

#### Douglas P. Mack^1,2^, Mariam A. Hanna^1,2^

##### ^1^Halton Pediatric Allergy, Burlington, Ontario, Canada; ^2^Department of Pediatrics, McMaster University, Hamilton, Ontario, Canada

###### **Correspondence:** Douglas P. Mack

*Allergy, Asthma & Clinical Immunology* 2018, **14(Suppl 1):**A41

**Background:** As the incidence of food allergies continues to rise, potential therapies are investigated. Oral immunotherapy (OIT) for food has been studied in the pediatric population for milk. We sought to investigate the use of oral milk immunotherapy to desensitize patients with milk allergy.

**Methods:** This is a retrospective review from 2014 to 2016 of OIT in a pediatric outpatient clinic ages 5–13 years (mean 9.8). After initial evaluation, testing, detailed informed consent/assent and potential oral challenge, participants were given the opportunity to be desensitized to milk starting with 1 drop of 1:25 diluted milk in sterile water. Maintenance target dose was 250 mL of cow’s milk. Doses were increased in clinic on a weekly—biweekly basis. A sublingual/oral immunotherapy approach was used. Those with asthma and prior severe reaction were not excluded.

**Results:** Oral desensitization was successfully completed in 4/5 patients initially enrolled. One participant withdrew because of personal reasons. The mean length of time to target dose was 31 weeks. The majority of individuals reported side effects, most of which were mild, some requiring antihistamine use, and no moderate or severe reactions were noted. All participants had experienced prior anaphylaxis before enrollment. Epinephrine was not administered during OIT. Mean initial skin test size was 9 mm (range 8–10). Skin prick testing became negative in the patients tested after being on maintenance therapy for 12 months.

**Conclusions:** This is the first Canadian study to demonstrate that oral immunotherapy can be safely and effectively used in a private practice setting for milk allergy in a pediatric population.

### A42 A case of tetany and osteopenia from vitamin D deficiency in an adolescent with cow’s milk allergy

#### Raymond Mak^1^, Kyla J. Hildebrand^1,2^

##### ^1^Division of Allergy and Immunology, Department of Medicine, University of British Columbia, Vancouver, British Columbia, Canada; ^2^BC Children’s Hospital Research Institute, Vancouver, British Columbia, Canada

###### **Correspondence:** Raymond Mak

*Allergy, Asthma & Clinical Immunology* 2018, **14(Suppl 1):**A42

**Background:** In Canada, biochemically low levels of vitamin D can be detected in 5.6% of children between ages 3–18 years. Important dietary sources of vitamin D include fortified milk, margarine, fish, red meats and liver. Children who consume cows’ milk regularly at least once daily are less likely to develop vitamin D deficiency. Severe vitamin D deficiency can result in hypocalcemia, osteopenia and Rickett’s.

**Clinical presentation:** Our patient had atopic dermatitis in infancy. At age three, extensive skin prick testing determined sensitization to multiple foods. The family subsequently avoided cow’s milk, fish, shellfish, eggs, peanut and tree nuts. As an alternative to milk, he drank mainly unfortified juices and water. No further allergy follow-up occurred.

At age 14 years, he presented to hospital with tetany in the upper and lower extremities. Investigations included: ionized calcium level = 0.60 mmol/L (1.1–1.3 mmol/L), parathyroid hormone level = 45.9 ng/L (1.5–7.6 ng/L), and vitamin D (25-OH) level < 10 nmol/L (75–200 nmol/L). Celiac disease and renal causes were excluded. A skeletal X-ray reported osteopenia. Allergy consultation determined an absence of past IgE-mediated reactions to any foods. Skin prick testing was positive to cow’s milk, egg, peanut, tree nuts and shellfish, and negative to fish.

**Management and discussion:** He received intravenous calcium, vitamin D supplementation (1000 IU/day) and a referral to a dietician. Oral food challenges are planned to clarify food allergy status. Potential long-term harm can occur when skin testing is performed at an early age without history of IgE-mediated reactions to foods, and when patients are lost to allergy follow-up. Sensitization alone does not indicate a food allergy. When age-appropriate, nutritionally replete substitutions are not offered, severe vitamin D deficiency can be a long-term sequelae of prolonged cow’s milk avoidance. Vitamin D supplementation (400 IU/day) should be considered in cow’s milk allergic individuals because of inadequate dairy intake.

**Consent to publish:** Written informed consent to publish was obtained from the patient’s guardians.

### A43 Prevalence of peanut allergy among high-risk infants with peanut introduction before and after assessment in Pediatric Allergy Clinic

#### Mihaela Paina^1^, Matthew Levesque^2^, Elinor Simons^1,2^

##### ^1^Section of Allergy and Clinical Immunology, Department of Pediatrics, University of Manitoba, Winnipeg, Manitoba, Canada; ^2^Children’s Hospital Research Institute of Manitoba, Department of Pediatrics, University of Manitoba, Winnipeg, Manitoba, Canada

###### **Correspondence:** Mihaela Paina

*Allergy, Asthma & Clinical Immunology* 2018, **14(Suppl 1):**A43

**Background:** In 2015, Allergy Consensus Guidelines began recommending peanut introduction before age 12 months for high-risk infants in countries with high peanut allergy prevalence. They suggested that these infants may benefit from an allergist’s assessment before peanut introduction. We evaluated if peanut allergy prevalence was different among infants who had peanut introduced before assessment versus infants who had peanut introduced after assessment.

**Methods:** We screened infants born in 2015 and 2016 who were referred to Pediatric Allergy Clinic before age 12 months, regardless of the reason for referral. Children at high risk for peanut allergy because of egg allergy and/or moderate-severe atopic dermatitis were included. We compared the prevalence of peanut allergy among infants who were first introduced to peanut before allergy assessment versus infants who were first introduced to peanut after assessment. Data were analyzed by Chi square and Kruskal–Wallis tests.

**Results:** Of the 42 infants at high risk for peanut allergy, peanut introduction data were available for 34: 13 (38.2%) had peanut introduced before allergy assessment, 13 (38.2%) had peanut introduced after assessment, and 8 (23.5%) did not have peanut introduced. Baseline characteristics were similar among the three groups: egg allergy (76.9, 61.5, and 50.0%, p = 0.21), moderate-severe atopic dermatitis (46.2, 76.9, and 75.0%, p = 0.14), age at first visit (11.1, 9.0, and 10.8 months, p = 0.31) and peanut sensitization (38.5, 46.2, and 50.0%, p = 0.60). For infants who had peanut introduced before and after allergy assessment, median age of peanut introduction was 8.0 versus 11.0 months (p = 0.017), peanut was introduced before age 12 months in 92.3% versus 61.5% (p = 0.068), and peanut allergy prevalence was 38.5% versus 38.5% (p = 1.0).

**Conclusions:** Children introduced to peanut after allergy assessment had peanut introduced later but were as likely to be introduced before age 12 months. Peanut allergy prevalence was similar among infants introduced before and after assessment.

### A44 Fair prioritization of patient referrals for oral immunotherapy in a public health system

#### Béatrice Paradis, Noémie Paradis, Jonathan Lacombe, Louis Paradis, Anne Des Roches, Philippe Bégin

##### Département de pédiatrie, CHU Ste-Justine, Montréal, Québec, Canada

###### **Correspondence:** Béatrice Paradis

*Allergy, Asthma & Clinical Immunology* 2018, **14(Suppl 1):**A44

**Background:** In medical ethics, the justice principle holds that when allocating care, patients in similar situations should have access to the same care. The recent opening of a publicly funded oral immunotherapy (OIT) program at our center, raised ethical issues as the demand for treatment was expected to significantly exceed its capacity. Criteria for patient selection had to be determined that would respect the justice principle and have high acceptability from patients, clinicians and managers.

**Methods:** Using a Delphi approach with 3 iterations, a panel of 25 experts were asked to propose and validate prioritization criteria for OIT, and establish their respective weights based on a fictive scenario. The consensual weights were used to determine prioritization ratios for patient subgroups and design a system that would adjust in real-time to the prevalence of these subgroups within the waiting list. This system was submitted to the expert for approval.

**Results:** The 25 experts that agreed to participate to the Delphi included 8 representatives of patient groups, 10 allergists (4 with experience with OIT), 3 allergy nurses, 2 administrative agents with experience managing tertiary care allergy referrals and 2 managers. In addition to validating the admissibility criteria, the participants reached a consensus on 4 main prioritization criteria and their prioritization ratio (PR):

- Allergy to multiple food groups (PR = 2.53).
- Allergy to an ubiquitary food in its cooked form (wheat, soy, milk, egg) (PR = 2.74).
- Age less than 3 years (PR = 2.74) and age 4–7 years (PR = 1.54).
- Food allergy-related quality of life judged within 10% (PR = 6.86), 25% (PR = 3.06) and 50% (PR = 1.72) worst of the cohort.

**Conclusions:** The system based on applying consensual PR to subgroup prevalence within waiting list was unanimously approved by a multidisciplinary expert group. The most frequently mentioned comment was that the PR system gave a real opportunity for less severe but admissible patients to benefit from the program, whereas traditional additive scoring system would de facto result in these patients never being seen, and were thus perceived as unfair.

### A45 Safety of daily food introduction in food allergic children with high reaction thresholds

#### Noémie Paradis^1^, Béatrice Paradis^1^, Louis Paradis^1,2^, Jonathan Lacombe^1^, Anne Des Roches^1^, Philippe Bégin^1,2^

##### ^1^Département de pédiatrie, CHU Ste-Justine, Montréal, Québec, Canada; ^2^Département de médecine, CHUM, Montréal, Québec, Canada

###### **Correspondence:** Noémie Paradis

*Allergy, Asthma & Clinical Immunology* 2018, **14(Suppl 1):**A45

**Background:** While oral immunotherapy (OIT) is a promising new approach to treat food allergies, it is associated with significant costs, deriving mostly from frequent up-dosing visits, individual food dose preparation and supervision of home dosing reactions. About half of food allergic children react to over 300 mg of allergen protein content, which would allow them to tolerate small amounts of the food in their daily diet. Such patients with spontaneous partial oral tolerance (SPOT) could potentially benefit from progressive introduction of their allergen in their diet without the burden of classical OIT approaches.

**Methods:** Charts from all patients with SPOT who underwent progressive allergen introduction after May 2012 were reviewed. Descriptive analysis was undertaken for patient demographic and clinical characteristics, reaction thresholds, food introduction dynamics (starting daily food dose and progression), and reaction rates at home and with up-dosing in the clinic.

**Results:** Ninety-five (95) patients aged 1–18 years with SPOT underwent progressive daily introduction of their allergen in their diet during the studied period. Index foods included egg (23%), soy (3%), wheat  (3%), milk (13%), sesame (3%), peanuts (46%) and tree nuts (8%). The average reactivity threshold was lowest for milk (725 mg of proteins) and highest for peanut (5763.78 mg of proteins). During an average follow-up of 1.5 years, the main symptoms reported by patients included oral symptoms (9%), urticaria (2%), eczema (2%), dyspepsia (2%) and cough (1%). No anaphylactic reaction was observed. Nine percent (9%) of patients had dose reduction at some point during the follow-up. At time of analysis, 80% of patients tolerated a daily dose that was higher than their initial reaction threshold. Patient satisfaction was very high.

**Conclusions:** Progressive introduction of food allergen at home is well tolerated and could be a less costly approach than traditional OIT in patient with SPOT.

### A46 Cross Canada Anaphylaxis Registry (CCARE): Comparing the epidemiology and management of anaphylaxis in Calgary and Montreal

#### Juan C. Ruiz^1^, Victoria Zotova^1^, Moshe Ben-Shoshan^2^, Yuka Asai^3^, Adil Adatia^4^, Yarden Yanishevsky^5^, Edmond S. Chan^6^, Greg Shand^7^, and Scott Delaney^8^, Ann Clarke^9^

##### ^1^Department of Medicine, Cumming School of Medicine, Calgary, Alberta, Canada; ^2^Division of Pediatric Allergy & Immunology, Department of Pediatrics, Montreal Children’s Hospital, Montreal, Quebec, Canada; ^3^Division of Dermatology, Department of Medicine, Queen’s University, Kingston, Ontario, Canada; ^4^Department of Medicine, University of Alberta, Edmonton, Alberta, Canada; ^5^Section of Allergy & Clinical Immunology, Department of Pediatrics, University of Alberta, Edmonton, Alberta, Canada; ^6^Division of Allergy & Immunology, Department of Pediatrics, Faculty of Medicine, University of British Columbia, BC Children’s Hospital, Vancouver, British Columbia, Canada; ^7^Division of Clinical Epidemiology, McGill University Health Centre, Montreal, Quebec, Canada; ^8^Department of Emergency Medicine, McGill University Health Centre, Montreal, Quebec, Canada; ^9^Division of Rheumatology, Cumming School of Medicine, Calgary, Alberta, Canada

###### **Correspondence:** Juan C. Ruiz

*Allergy, Asthma & Clinical Immunology* 2018, **14(Suppl 1):**A46

**Background:** To compare the rates, triggers, and management of anaphylaxis in adult emergency departments (ED) in Calgary and Montreal.

**Methods:** As part of the Cross-Canada Anaphylaxis Registry (C-CARE), anaphylaxis cases fulfilling the consensus definition were identified through chart review of adults presenting with anaphylaxis and allergy-related ICD-10 codes to a Calgary tertiary care ED in 2015. These cases were compared with published data on similarly identified cases from a Montreal ED in 2011.

**Results:** Among 79,459 presentations to the Calgary ED, 112 had anaphylaxis, yielding a prevalence of 0.14% (95% CI 0.12%, 0.17%). The most common trigger was food 63.4% (95% CI 53.8%, 72.3%). Among food-induced cases, peanuts triggered 22.5% (95% CI 13.5%, 34.0%), tree nuts 16.9% (95% CI 9.1%, 27.7%), milk 9.9% (95% CI 4.1%, 19.3%), and shellfish 8.5% (95% CI 3.2%, 17.5%). Drugs triggered 14.3% (95% CI 8.4%, 22.2%) of cases. Epinephrine was administered to 81.3% (95% CI 72.8%, 88.0%) of patients and 81.3% (95% CI 71.0%, 89.1%) were prescribed an auto-injector.In Montreal, 0.26% (95% CI 0.21%, 0.32%) of ED presentations were due to anaphylaxis. 63.3% (95% CI 52.9%, 72.6%) were triggered by food, primarily shellfish [12.9% (95% CI 6.1%, 24.4%)] and peanuts [8.1% (95% CI 3.0%, 18.5%)], and 18.4% (95% CI 11.5%, 27.7%) by drugs. Epinephrine was used in 49.0% (95% CI 38.8%, 59.2%) and 67.1% (95% CI 55.3%, 77.2%) were prescribed an auto-injector.

**Conclusions:** Calgary has a lower rate of anaphylaxis than Montreal (difference − 0.12% (95% CI − 0.18%, − 0.06%). Food was responsible for 63% of cases in both sites. Peanut was the most common food allergen in Calgary versus shellfish in Montreal. Shellfish and dust mite allergy are strongly associated, and the absence of dust mites in Calgary potentially results in less shellfish allergy. Epinephrine administration was higher in Calgary, possibly because the Calgary study was conducted 4 years later and reflects increased awareness about anaphylaxis management.

### A47 PD-L1^+^ regulatory B cells increase during milk oral immunotherapy

#### Bahar Torabi^1^, Marieme Dembele^1^, Duncan Lejtenyi^1^, Moshe Ben-Shoshan^1,2^, Bruce D Mazer^1,2^

##### ^1^Research Institute of the McGill University Health Centre, Montreal, Quebec, Canada; ^2^Division of Pediatric Allergy and Clinical Immunology, Montreal Children’s Hospital, McGill University, Montreal, Quebec, Canada

###### **Correspondence:** Bahar Torabi

*Allergy, Asthma & Clinical Immunology* 2018, **14(Suppl 1):**A47

**Introduction:** Regulatory B-cells (Bregs) have been implicated in venom immunotherapy and their role is being actively studied in non-IgE-mediated food allergies and autoimmune diseases. No studies have examined the correlation between Bregs and IgE-mediated milk allergy, nor have the action of Bregs been examined in the treatment of food allergies with oral immunotherapy (OIT). Furthermore, there are currently no phenotypic, transcription factor, or lineage markers unique to regulatory B cells, making it a diverse and challenging focus of research. Programmed death-ligand 1 (PD-L1) is one of the surface molecules described on Bregs. PD-L1 is an inhibitory ligand expressed on antigen-presenting cells and tumor cells.

**Methods:** Peripheral blood mononuclear cells were isolated from plasma of milk-allergic children undergoing milk OIT, at baseline and at the end of escalation phase. The cells were cultured for 72 h in various conditions and stained for CD19, CD27, CD38, CD5, CD24, PD-L1, and intracellular IL-10. Conditions included CpG-B, anti-IgM/IgG, and anti-CD40, IL4, IL21. Milk proteins (casein, α-lactalbumin, β-lactoglobulin) were added to some conditions as specific antigens. Statistical analysis was done using the Wilcoxon matched-pairs signed rank test and a p-value less than 0.05 was considered significant.

**Results:** An interim analysis showed a significant increase in 6 patients in the CD19^dim^PD-L1^+^CD38^+^ population at the end of escalation phase in 3 conditions: CpG-B/anti-IgM/IgG/anti-CD40, anti-CD40/IL4/IL21, and anti-CD40/IL4/IL21 plus milk proteins. The median percentage difference between baseline and end of escalation phase was 8.35, 4.49, and 7.12% for the above conditions, respectively. The majority (89.16%, 95% CI 81.21–95.56%) of the CD19^dim^PD-L1^+^ population in all conditions were CD38^+^ cells.

**Conclusions:** PD-L^+^ regulatory B cells increase during milk OIT and may be part of the mechanism of successful desensitization in children. This population of Bregs could play a role in other allergic diseases as well. Further assessment with a larger sample size is underway.

### A48 Venom triggered anaphylaxis cases management and clinical characteristics in Canada

#### Sarah Zahabi^1^, Ann Clarke^2^, Sofianne Gabrielli^3^, Jocelyn Moissan^4^, Harley Eisman^5^, Judy Morris^6^, Edmond S. Chan^7^, Greg Shand^8^, Moshe-Ben-Shoshan^9^

##### ^1^McGill University, Montreal, Quebec, Canada; ^2^Division of Rheumatology, Department of Medicine, Cumming School of Medicine, University of Calgary, Calgary, Alberta, Canada; ^3^Division of Pediatric Allergy and Clinical Immunology, Department of Pediatrics, McGill University Health Centre, Montreal, Quebec, Canada; ^4^Regional Medical director of Emergency Medical Services of Outaouais, Outaouais, Quebec, Canada; ^5^Department of Emergency Medicine, Montreal Children’s Hospital, McGill University Health Centre, Montreal, Quebec, Canada; ^6^Department of Emergency Medicine, Sacre-Coeur Hospital, Montreal, Quebec, Canada; ^7^Division of Allergy and Immunology, Department of Pediatrics, BC Children’s Hospital, University of British Columbia, Vancouver, British Columbia, Canada; ^8^The Research Institute of the McGill University Health Centre, Meakins-Christie Laboratories, Division of Pediatric Allergy and Clinical Immunology, Department of Pediatrics, Montreal Children’s Hospital, Montreal, Quebec, Canada; ^9^Division of Pediatric Allergy and Clinical Immunology, Department of Pediatrics, McGill University Health Centre, Montreal, Quebec, Canada

###### **Correspondence:** Sarah Zahabi

*Allergy, Asthma & Clinical Immunology* 2018, **14(Suppl 1):**A48

**Background:** Venom triggered anaphylaxis cases (VTAC) represent 4.1% of adult anaphylactic reactions and account for the most severe reactions. The aim of this study was to collect data on patient characteristics associated with VTAC and to compare the management of these cases in both the pre-hospital and emergency department (ED) setting in Canada to the clinical guidelines.

**Methods:** The Cross-Canada Anaphylaxis Registry (C-CARE) enrolls anaphylactic cases presenting to EDs and out-of-hospital emergency medical services (EMS). We utilized C-CARE to identify VTAC that presented to EDs at the Montreal Children’s Hospital and Sacré-Coeur Hospital, and to EMS in Western Quebec from June 2013 to May 2017. ED physicians and EMS paramedics documented characteristics, triggers, and management using standardized forms. Consenting patients were contacted annually regarding long-term management. Univariate and multivariate logistic regression were used to identify factors associated with epinephrine treatment and severe reactions.

**Results:** Between June 2013 and May 2017, 115 VTAC were identified, of which 60% were prospectively recruited. Epinephrine was administered to 63.5% (95% CI 53.9, 72.1%) of all VTAC by a healthcare professional. Reactions occurring at home were more likely managed without epinephrine (OR 1.27 [95% CI, 1.09, 1.47]). Severe reactions were more likely to occur in patients receiving regular daily nonsteroidal anti-inflammatory drug treatment (OR 1.45 [95% CI, 1.03, 2.07]) and while at home (OR 1.25 [95% CI, 1.04, 1.49]) while controlling for age, beta-blocker use, reactions occurring during exercise and administration of anti-histamines in-hospital. Limited follow-up data was obtained to due low response rate; however 29 patients were successfully contacted of which 89.7% (95% CI, 71.5%, 97.3%) were prescribed an epinephrine auto-injector, 44.8% (95% CI, 27.0%, 64.0%) saw an allergist who confirmed the allergy, and 27.6% (95% CI, 13.4%, 47.5%) received immunotherapy.

**Conclusions:** Clinical guidelines strongly encourage that VTAC be referred to an allergist for confirmation of allergy and desensitization treatment, however less than 50% of patients had confirmation and less than a third of those confirmed received immunotherapy. Given that the guidelines are not adhered to in clinical practice, several knowledge translation solutions should be explored and implemented in order to close this knowledge-to-action gap.

## Basic Science/Immunology

### A49 Expression profile of IL-33 in the skin of atopic dermatitis post-allergen exposure

#### Dhuha Al-Sajee, Emma Price, Heidi Yin, Karen Howie, Paul M. O’Byrne, Hermenio Lima, Gail M. Gauvreau, Roma Sehmi

##### Department of Medicine, McMaster University, Hamilton, Ontario, Canada

###### **Correspondence:** Dhuha Al-Sajee

*Allergy, Asthma & Clinical Immunology* 2018, **14(Suppl 1):**A49

**Background:** The barrier-derived cytokine Interleukin-33 (IL-33) is a critical regulator of pathological processes in atopic dermatitis. This alarmin signals through interleukin-1 receptor-like-1 (ST2) receptor resulting in a Th2 immune response. We examined the expression of IL-33 and ST2 in skin following an intradermal allergen challenge in patients with atopic dermatitis.

**Methods:** Patients with moderate to severe atopic dermatitis who developed a late cutaneous response to intradermal challenge, with common airborne allergens were recruited. Patients withheld oral steroid (8 days) and other immunosuppressive medications (4 weeks) prior to challenge. Allergen and saline control were injected intradermally on the patient’s back. At 24 h post-challenge, the size of the wheel and flare was measured and a punch biopsy was obtained. Biopsy tissue was formalin-fixed, paraffin embedded, and stained with antibodies to IL-33 and ST2 for immunofluorescence microscopy. Regions of interest (ROI) highlighting the epidermis and dermis were selected and images were obtained using an upright Nikon microscope. The thresholding tool in the NIS-element software was set to pick up the radius of 6 pixels from selected channels corresponding to the intensity value of these pixels. All pixels in the image of similar intensity value were highlighted. Data was exported to excel showing the number of objects and the mean intensity of each channel in the selected ROI.

**Results:** Data are presented for 4 patients. When compared to saline, intradermal allergen increased the number of cells/mm^2^ expressing IL-33 by approximately 30% in the dermis (p = 0.02) and epidermis + dermis (p = 0.03). In the epidermis, the number of cells/mm^2^ expressing IL-33 increased in 3 of the 4 patients studied (p = 0.09). No significant changes in ST2 expression were observed, due to the small sample size.

**Conclusions:** Our data supports the role of IL-33 in acute allergen-induced inflammatory processes in atopic dermatitis.

### A50 Bicyclic petasite sesquiterpenes potentiate peroxisome proliferator activated receptor gamma activator and inhibit dendritic cell maturation and activation

#### Narcy Arizmendi^1^, Yiming Li^2^, Fujiang Guo^2^, Marianna Kulka^1,3^

##### ^1^National Institute for Nanotechnology, National Research Council Canada, Edmonton, Alberta, Canada; ^2^School of Pharmacy, Shanghai University of Traditional Chinese Medicine Shanghai, Shanghai Shi, China; ^3^Department of Medical Microbiology and Immunology, University of Alberta, Edmonton, Alberta, Canada

###### **Correspondence:** Narcy Arizmendi

*Allergy, Asthma & Clinical Immunology* 2018, **14(Suppl 1):**A50

**Background:** The anti-inflammatory role of many plant derivatives is not fully understood. There is evidence that a family of plant derivatives, the petasite sesquiterpenes, can regulate the immune system through dendritic cell (DC) targeting. DC activation induces their expression of class-II major histocompatibility complex (MHC) and co-stimulatory surface molecules, as well as migration into secondary lymphoid organs, where they activate naïve T-cells. To better understand the anti-inflammatory effects of petasite sesquiterpenes, we focused on their activation mechanism involving peroxisome proliferator activated receptor gamma (PPARγ). PPARγ is a transcription factor that inhibits inflammatory responses and induces DCs to acquire a mucosal phenotype. Since mucosal DCs are central in innate immune responses, we hypothesized that sesquiterpenes would inhibit DC maturation and activation via the PPARγ pathway.

**Methods:** We generated mouse bone marrow-derived DC (BmDC) in media supplemented with GM-CSF+IL-4. BmDC were treated with bicyclic petasite sesquiterpenes in the presence or absence of PPARγ agonists, followed by overnight activation with LPS. BmDC were harvested and analyzed for surface expression of co-stimulatory molecules CD80 and CD86, by flow cytometry, and pro-inflammatory cytokine production, by commercial ELISA.

**Results:** Bicyclic petasite sesquiterpenes downregulated the expression of the co-stimulatory molecules CD80 and CD86 and proinflammatory cytokines of LPS-activated DCs. We observed differences in the downregulation of CD80 and CD86, and in TNF, IL-6 and IL-12p70 release when LPS-activated DC were pretreated with petasite sesquiterpenes or combinations of petasite sesquiterpenes and PPARγ agonists.

**Conclusions:** Bicyclic petasite sesquiterpenes inhibited the maturation and activation of DC, and this inhibitory activity was enhanced in the presence of PPARγ agonists. Studies are underway to determine the intermediary molecules involved in DC inhibition.

### A52 Examining the impact of TLR2 and cow’s milk on oral tolerance development

#### Bassel Dawod^1,3^, Ian D. Haidl^2,3^, Alexander J. Edgar^2,3^, Matthew C. Tunis^2,3^, Jean S. Marshall^1,2,3^

##### ^1^Department of Pathology, Dalhousie University, Halifax, Nova Scotia, Canada; ^2^Department of Microbiology & Immunology, Dalhousie University, Halifax, Nova Scotia, Canada; ^3^Dalhousie Inflammation Group, Dalhousie University, Halifax, Nova Scotia, Canada

###### **Correspondence:** Bassel Dawod

*Allergy, Asthma & Clinical Immunology* 2018, **14(Suppl 1):**A52

**Background:** Breast milk feeding has many beneficial effects, including a reduced risk of food allergy. Cow’s milk also contains several regulatory molecules, such as TGF-β and vitamin A, which could enhance oral tolerance. Toll-like receptor 2 (TLR2) activation by pathogen products or their analogues can lead to tolerance disruption. Soluble toll-like receptor 2 (sTLR2) acts as a decoy receptor and is found in both human and cow’s milk; however, its role in oral tolerance is unknown.

**Hypothesis:** We hypothesize that sTLR2 can block tolerance disruption by TLR2 ligands found in food or microbes.

**Methods:** Oral tolerance was assessed in wild-type and TLR2-deficient mice through analysis of antigen-specific antibody responses after OVA feeding and a systemic antigen challenge. The ability of cow’s milk to block the anti-tolerogenic effects of a TLR2 activator (PAM3CSK4) was assessed. Tolerance to OVA was also assessed in mice that were nursed by WT or TLR2^−/−^ dams. The impact of cow`s milk on development of antigen-specific Treg cells and tolerogenic dendritic cells was also determined.

**Results:** Oral administration of antigens over 1 week induced the development of tolerance, independent of TLR2 expression. Tolerized mice produced 5.8-fold less allergen-specific IgE than controls (n = 15). Soluble TLR2 was detected in cow’s milk and in commercial baby formulas (range 15–35 ng/mL). Interestingly, cross-fostering of pups by WT dams resulting better oral tolerance induction to OVA compared to pups nursed by TLR2^−/−^ dams. Furthermore, cow’s milk feeding was also able to enhance the development of ovalbumin-specific Treg cells and tolerogenic-dendritic cells significantly in Peyer’s patches and mesenteric lymph nodes of mice.

**Conclusions:** Our results suggest an important role for cow’s milk-regulatory molecules and sTLR2 in the development of oral tolerance, which could inform both allergy prevention and treatment strategies.This work was supported by CIHR and Allergen N.C.E

### A54 Anti-neuroinflammatory response of hesperetin on BV-2 murine microgila

#### Mi Eun Kim, Sunhyo Jo, Jun Sik Lee

##### Department of Biology and BK21-plus Research Team for Bioactive Control Technology, College of Natural Science, Chosun University, Gwangju 501-759, South Korea

###### **Correspondence:** Mi Eun Kim

*Allergy, Asthma & Clinical Immunology* 2018, **14(Suppl 1):**A54

**Background:** Neuroinflammation is a specific immunological reaction in the central nerve system and is distinct from peripheral inflammatory responses. It is induced by microglia, which is a resident phagocyte in the brain. Neuroinflammatory response is a defense system against exogenous antigens, but chronic neuroinflammation is involved in various neurodegenerative disorders. Hesperetin is present in the peel of citrus as one of the flavonones. In previous studies, hesperetin has been shown to have anti-inflammation, anti-cancer and anti-oxidant effects. However, the anti-neuroinflammatory effect of hesperetin on microglia is not well known.

**Methods:** BV-2 murine microglia cell line was used in this study. Cytotoxicity of heperitin on BV-2 was measured by MTT assay and NO production was evaluated by Griess assay. Expression of pro-inflammatory genes and signal transduction protein were determined by RT-PCR and western blot analysis, respectively. Cytokine release was determined by ELISA.

**Results:** Heperetin reduced the LPS-induced no production at non-cytotoxic concentration. Furthermore, hesperetin effectively inhibits mRNA expression of pro-inflammatory molecules including TNF-α, iNOS, and IL-6 and IL-1β, and cytokines, IL-6. Hesperetin inhibited the phophorylation of p38 and ERK in LPS-activated BV-2 microglia.

**Conclusions:** These results suggest that hesperetin has an anti-neuroinflammatory effect on LPS-activated BV-2 murine microglia through the suppression of p38 and ERK phosphorylation.

### A55 Mast cells enhance resistance against influenza A in epithelial cells

#### Kurtis Ng, Tae Chul Moon, Harissios Vliagoftis, Dean Befus

##### Department of Medicine, University of Alberta, Edmonton, Alberta, Canada

###### **Correspondence:** Kurtis Ng

*Allergy, Asthma & Clinical Immunology* 2018, **14(Suppl 1):**A55

**Background:** The role of mast cells (MC) in host defences against influenza A virus (FluA) is incompletely understood. We found that MC were resistant to productive FluA infection and suppress viral propagation in airway epithelial cells (AEC) in co-culture systems. We hypothesized that MC interact with AEC to produce a factor(s) that enhances anti-viral capabilities, therefore reducing viral replication and release of infectious particles and enhancing AEC survival.

**Methods:** Our experimental model to investigate MC and AEC interaction during FluA infection involves a co-culture system with AEC (Calu-3) cultured on the membrane insert in the top chamber, and human MC (LAD2) in the bottom chamber. FluA infection was achieved by exposing AEC to A/PR/8/34 (H1N1; 0.04 MOI [multiplicity of infection]). Hemagglutination assay was used to assess viral propagation 3 days post infection. Supernatants of post-FluA experienced AEC-MC co-cultures were collected for further investigation. Supernatants were treated to 65 and 100 °C for 30 min to investigate heat lability of anti-viral activity. Fractionation of supernatants were performed using Centricon size exclusion filters (10 kDa). Ion exchange columns are used to further fractionate supernatant by molecular charge.

**Results:** In the presence of MC, AEC were found to release 3-fold less FluA particles. Supernatant from a FluA experienced AEC-MC co-culture exhibits an antiviral effect against FluA in AEC. Heat treatment of the supernatant suggest that the antiviral effect is sensitive to 100 °C but not 65 °C. Fractionation approaches suggest that the antiviral effect is larger than 10 kDa. Preliminary data from ion exchange suggest that anti-viral activity has a high net positive charge.

**Conclusions:** FluA experienced co-culture supernatant has anti-viral activity mediated through a soluble factor(s) against FluA infection in AEC. Identification of the anti-viral factor(s) may foster novel approaches to enhancing FluA vaccination and therapeutic strategies.

### A57 Incidence of primary immune deficiency in Canada, 2016

#### Robert B Hopkins^1,2^, Natasha Burke^1,2^, Antonio Giulivi^3^, David Barnes^4^, Ayman Kafal^4^, Daria O’Reilly^1,2^

##### ^1^Department of Health Research Methods, Evidence, and Impact, McMaster University, Hamilton, Ontario, Canada; ^2^Programs for Assessment of Technology in Health (PATH), Research Institute of St. Joseph’s Hamilton, Hamilton, Ontario, Canada; ^3^Department of Pathology and Laboratory Medicine, Faculty of Medicine, University of Ottawa, Ottawa, Ontario, Canada; ^4^CSL Behring, Ottawa, Ontario, Canada

###### **Correspondence:** Daria O’Reilly

*Allergy, Asthma & Clinical Immunology* 2018, **14(Suppl 1):**A57

**Background:** Primary Immune Deficiency (PID) diseases constitute a group of disorders, usually genetic, that cause a malfunction in all or part of the immune system, thereby rendering the patient unable to fight off infections caused by everyday germs. Limited data on the epidemiology of PID is available for Canada. The objective was to estimate the incidence of PID in Canada in 2016.

**Methods:** We used mandatory standardized national administrative data from 10 fiscal years FY2006/2007 to FY2015/2016 to identify PID cases from acute care admissions, emergency visits, day surgery and hospital-based clinics. Incidence is defined as the presence of a PID ICD-10-CA code of D80-D84, D89.3, or D89.8 in FY2015/2016 without any PID code in the previous 9 years. Data coverage varies by province, and results are reported separately for provinces with admission and day surgery data only (all provinces except Quebec and British Columbia), for Ontario which additionally has emergency room visit data, and for Alberta which additionally has emergency and hospital-based medical clinics data.

**Results:** In Canada, in FY2015/2016, the incidence of PID based on admissions and day surgery was 3.4/100,000 population. After adding emergency room visits, the incidence was 4.0/100,000 population. Adding medical clinics data increased the incidence to 13.9/100,000 population (15.7 women, 12.2 men) which when extrapolated to the Canadian population represents 5354 new cases of PID annually. The average age of incidence was 44.5 years (standard deviation 26 years), with 53% women.

**Conclusions:** Using a robust approach for data collection, this study provides previously unavailable estimates of the incidence of PID in Canada, which were higher than expected. This may have important implications for treatment of PID, as there is a lifelong risk of recurrent infections which may lead to permanent organ damage and reduced life expectancy.

### A58 The novel characterization of eosinophil progenitors in the skin of atopic dermatitis patients following intradermal allergen challenge

#### Emma Price, Dhuha Al-Sajee, Sai Sakktee Krisna, Michael Aw, Michelle Yee, Karen Howie^1^, Caroline Munoz, Paul M. O’Byrne, Hermenio Lima, Roma Sehmi, Gail M Gauvreau

##### Department of Medicine, McMaster University, Hamilton, Ontario, Canada

###### **Correspondence:** Emma Price

*Allergy, Asthma & Clinical Immunology* 2018, **14(Suppl 1):**A58

**Background:** In atopic dermatitis (AD), systemic and local eosinophilia is thought to contribute to disease initiation and progression. In chronic allergic inflammatory diseases, tissue eosinophilia may arise from recruitment of mature cells in response to local chemoattractant production such as eotaxin, or through in situ differentiation of eosinophil lineage-committed progenitors (EoP), driven by growth factors such as IL-5. We have previously reported this latter mechanism in subjects with atopic airway diseases. Currently, little is known about the presence of EoP in the skin of patients with AD, or the importance of these cells in the pathogenesis of AD. This study aims to quantify EoP in the allergen induced late cutaneous response.

**Methods:** Subjects with severe AD and a positive allergen prick test were recruited (n = 4). After 8 days of systemic steroid washout, intradermal challenge was performed with allergen and saline control. The late cutaneous response was measured at 24 h post challenge and biopsied. A 4 mm skin biopsy was formalin-fixed, paraffin embedded and stained with hematoxylin and eosin for eosinophils. Immunofluorescence staining was performed using fluorochrome linked antibodies to CD34-FITC, IL-5Rα-Cy5, and Von Willebrand factor-TRITC. Using Nikon Imagine Software Analysis, the papillary dermis was examined for EoP (CD34+/IL-5Rα+) cells that were Von Willebrand negative. A paired t-test was performed on the data.

**Results:** We identified the presence of both CD34+/IL-5Rα+ cells and mature eosinophils in skin biopsies with perivascular organization. There was a significant increase in EoP (p < 0.05) and mature eosinophils (p < 0.05) 24 h post-allergen compared to diluent control.

**Conclusions:** Intradermal allergen challenge induces an increase in eosinophil lineage-committed progenitor cells and mature eosinophils in atopic dermatitis skin. Local in situ maturation may play a role in development of tissue eosinophilia in patients with atopic dermatitis.

## Other Allergy/Immunology

### A60 Omalizumab in the treatment of chronic inducible urticaria: experience from the allergy and immunology clinic at Centre Hospitalier de l’Université de Montréal (CHUM)

#### Catherine Besner Morin^1^, Hugo Chapdelaine^2^

##### ^1^Department of Dermatology, Université de Montréal, Montreal, Quebec, Canada; ^2^Department of Allergy and Immunology, Université de Montréal, Montreal, Quebec, Canada

###### **Correspondence:** Catherine Besner Morin

*Allergy, Asthma & Clinical Immunology* 2018, **14(Suppl 1):**A60

**Background:** Omalizumab is an anti-immunoglobulin E approved for the treatment of chronic spontaneous urticaria (CSU). Although its efficacy profile against CSU has been well documented in the literature, fewer reports have focused on chronic inducible urticarial (CindU).

**Methods:** Patient charts were reviewed at the allergy and immunology department of the University of Montreal for cases of urticarial treated with Omalizumab. In this patient population with a diagnosis of chronic urticarial, only 8 corresponded to CindU and were included in this case series. Patients were started at a dosage of 300 mg every 4 weeks with further adjustments of dosage or frequency depending on the symptomology reported during follow-up visits. Response to treatment was graded with the Urticaria Activity Score (UAS7) ranging from 0 to 42 and Chronic Urticaria-Quality of Life (CU-QoL) ranging from 23 to 115.

**Results:** CindU cases presented in different forms (cold, delayed pressure, solar, dermographism, cholinergic). From 8 patients, 3 remained on the initial treatment regimen, 1 was allowed to lower the dose to 150 mg and 4 required an increase in dosage or frequency, of which 3 received more than 600 mg. UAS7 scores ranged from 0 to 11 with a mean of 3.9 and a median of 2.0. CU-QoL scores ranged from 23 to 34, with a mean of 27.3 and a median of 25.5. When results were considered in function of the modified physician global assessment, 5 patients measured as clear to almost clear and 3 as partial responders. No patients showed limited or no response to treatment. Side effects were minimal.

**Conclusions:** In conclusion, we report 8 cases with various types of chronic inducible urticarial that safely and satisfactorily responded to Omalizumab. Dosage and frequency were adapted to patients’ symptomology. This flexibility helped patients to achieve complete to partial response to treatment.

### A61 Rush and ultrarush venom immunotherapy in very young children

#### Ana Copaescu^1,2^, François Graham^1,2^, Philippe Bégin^1,2^, Jonathan Lacombe-Barrios^2^, Kathryn Samaan^2^, Jean Paradis^1^, Louis Paradis^1,2^, Anne Des Roches^2^

##### ^1^Allergy and Immunology, Centre Hospitalier de l’Université de Montréal, Hôpital Notre-Dame, Montreal, Quebec, Canada; ^2^Allergy and Immunology, Centre Hospitalier Universitaire Sainte-Justine, Montreal, Quebec, Canada

###### **Correspondence:** Ana Copaescu

*Allergy, Asthma & Clinical Immunology* 2018, **14(Suppl 1):**A61

**Background:** Hymenoptera stings are a major cause of fatal anaphylaxis in children. Accelerated venom immunotherapy (VIT) schedules are more convenient and offer faster protection than conventional VIT. Although well studied in older children and adults, safety data in very young children is sparse.

**Methods:** Between 2013 and 2016, 12 patients aged 16 months to 6 years received rush (n = 6) or ultrarush (n = 6) VIT at the allergy clinic of the Ste-Justine Hospital Center. Patients’ mean age was 5 years, and 9 of the 12 (75%) were boys. At initial insect sting, 1 patient (8%) had a grade 1 reaction, 9 (75%) had grade 2 reactions, and 2 (17%) had grade 3 reactions as per Brown’s criteria. All patients had at least one positive skin test to standardized venom extracts of bee, wasp, yellow jacket, yellow hornet, and white face-hornet (Jubilant HollisterStier LLC, Spokane, Washington, USA). Four out of 12 (33%) were administered rush or ultrarush VIT to mixed vespid, 6 of the 12 (50%) to mixed vespid and wasp, and 2 of the 12 (17%) to bee venom. We systematically premedicated all patients with antihistamines 1 h before the desensitization. Patients undergoing the ultrarush protocol were additionally premedicated with prednisone (1 mg/kg) to reduce delayed local reactions. All of the children were observed up to 2 h after the last injection.

**Results:** Out of the 12 patients, none had any systemic reactions during buildup and maintenance doses. Only local reactions less than 10 cm were reported. One patient was restung and did not present any reaction during maintenance doses.

**Conclusions:** Although this cohort represents a small amount of patients under the age of 6, they support the conclusion that rush and ultrarush VIT schedules are safe in this very young age group.

### A62 Rituximab desensitizations: the Halifax experience

#### Pascale Dupuis^1^, Lori A. Connors^1,2^, Gina A. Lacuesta^2^

##### ^1^Department of Medicine, Dalhousie University, Halifax, Nova Scotia, Canada; ^2^Halifax Allergy and Asthma Associates, Halifax, Nova Scotia, Canada

###### **Correspondence:** Pascale Dupuis

*Allergy, Asthma & Clinical Immunology* 2018, **14(Suppl 1):**A62

**Background:** Rituximab is a monoclonal antibody targeted at CD20. It is used in chemotherapeutic regimens in several malignancies, as well as in a variety of autoimmune diseases, given its effects on B cells. Often, if a patient develops allergy to rituximab there are no alternative treatments, or the alternative is not as effective. There are published protocols for rituximab desensitization available but we were not able to find any case reports of their use in Canada.

**Case presentation:** Patient 1 is a 29 year old female with a longstanding history of systemic lupus erythematosus (SLE), complicated with hypertension and significant lupus nephritis. The patient had failed multiple therapeutics for SLE and was doing well on rituximab, until she developed Type 1 and Type III hypersensitivity to the drug. With pre and post-medications she was successfully desensitized to rituximab, using a 12-step protocol.

Patient 2 is a 70 year old female who presented with urticaria and respiratory symptoms during rituximab infusion for management of eosinophilic granulomatosis with polyangiitis. The patient was successfully desensitized to rituximab, using a 12-step protocol. Both patients were managed in an Intermediate Care setting as inpatients.

**Discussion:** In our patients, rapid desensitization to rituximab was well tolerated. The protocols were successfully completed. For one patient, desensitization was successful in preventing Type I and Type III hypersensitivity. For one patient, the first desensitization produced mild symptoms. Subsequent desensitizations have not produced any significant reactions. There are some challenges in arranging inpatient beds for these desensitizations, which has affected the timeliness of their drug dosing. A multidisciplinary approach was necessary to establish a protocol for our institution.

**Conclusions:** Rituximab desensitization can be safely done in a monitored setting with close supervision by an allergist.

**Consent:** Written consent was obtained from both individuals involved.

### A63 An in depth look at the medical management of hereditary angioedema through a Canadian

#### Lisa Fu^1^, Amin Kanani^2^, Gina Lacuesta^3^, Susan Waserman^4^, Stephen Betschel^1^

##### ^1^Department of Medicine, Division of Clinical Immunology and Allergy, University of Toronto, Toronto, Ontario, Canada; ^2^Division of Allergy and Immunology, Department of Medicine, University of British Columbia, Vancouver, British Columbia, Canada; ^3^Department of Medicine, Dalhousie University, Halifax, Nova Scotia, Canada; ^4^Division of Clinical Immunology Allergy, McMaster University, Hamilton, Ontario, Canada

###### **Correspondence:** Lisa Fu

*Allergy, Asthma & Clinical Immunology* 2018, **14(Suppl 1):**A63

**Background:** Hereditary angioedema (HAE) is a rare disease that has significant morbidity and may be potentially fatal due to airway obstruction. Our study aimed to determine how Canadian physicians diagnose and treat HAE.

**Methods:** A survey was designed to determine HAE practice patterns amongst Canadian physicians. These physicians were identified by sending the survey to members of three physician organizations: the Canadian Hereditary Angioedema Network, the Canadian Society of Clinical Immunology and Allergy, and the Canadian Hematology Society.

**Results:** Thirty-six physicians responded to the survey. Thirty-four physicians were included in the analysis. The majority of referrals to HAE treating physicians were from family and emergency room physicians. The most common sites of swelling reported by patients to physicians were facial, peripheral and abdominal. A mean of 53.9% of HAE-Type 1 and II patients and 53.4% of HAEnC1INH patients were on long term prophylaxis. A mean of 41.9, 19.4 and 93.5% of respondents had some patients on danazol, tranexamic acid and C1-inhibitor respectively. The majority of physicians felt severity and frequency of attacks were the most important determinants in deciding when to use prophylaxis. A mean of 88.2% of physicians used C1-inhibitor to treat acute attacks and 79.4% used icatibant. A mean of 91.4 and 94.3% of respondents felt very or extremely confident using C1-inhibitor for prophylaxis and for acute attacks respectively. A mean of 71.4% of physicians felt very or extremely confident in using icatibant. All respondents were aware of HAE guidelines. Respondents felt a further need for guidance when managing HAE in pregnancy, and pediatric patients.

**Conclusions:** Physicians are using guidelines to support their practice, and using agents suggested by guidelines with confidence. C1-inhibitor is being used widely for prophylaxis, as well as acute treatment of attacks along with icatibant. However, certain special patient populations may require additional focus in future guidelines.

### A64 Diagnosis of macrolide allergy through graded oral challenge

#### Sofianne Gabrielli^1^, Bahar Torabi^1^, Christine Lejtenyi^1^, Elaine Medoff^1^, Moshe Ben-Shoshan^1^

##### ^1^Division of Pediatric Allergy and Clinical Immunology, Department of Pediatrics, McGill University Health Centre, Montreal, Quebec, Canada

###### **Correspondence:** Sofianne Gabrielli

*Allergy, Asthma & Clinical Immunology* 2018, **14(Suppl 1):**A64

**Background:** Macrolides are commonly used antibiotics in children, however there are no standardized skin tests available for diagnosis of allergy. While up to 3% of children report reactions to macrolides, it is not clear how many have a true allergy. Our aim was to assess the risk of macrolide allergy in patients through the use of a graded oral challenge.

**Methods:** All children referred to the Montreal Children’s Hospital for potential antibiotic allergy were recruited for the LAACTAM study between March 2013 and 2017. A standardized survey with questions on treatment, symptoms, and associated factors was filled and an oral challenge (10 and 90% of the oral dose) was conducted at the clinic visit. The patients were contacted annually to query on subsequent antibiotic use and associated reactions. Multivariate logistic regression was used to estimate factors associated with a positive oral challenge.

**Results:** Seventy-seven patients with a reported allergy to macrolide antibiotics were recruited, with 79.2% (95% CI, 71.4%, 88.5%) reporting a reaction to clarithromycin and 19.5% (95% CI, 11.7%, 28.7%) to azithromycin. The majority of the reactions were isolated to the skin and occurred within 3 days of starting treatment. Among 68 patients who underwent an oral challenge, 3 patients (4.4% [95% CI, 1.5%, 9.4%]) had a positive challenge, of which all were immediate reactions.

A positive oral challenge to a macrolide antibiotic was associated with older age (OR 1.03 [95% CI, 1.01, 1.04]) while adjusting for sex, type of macrolide, history of known food allergy, drug allergy, and asthma.

Among the 65 patients with negative oral challenge eligible for follow-up, 48 (73.8%) patients responded. Of the contacted patients, 15 (31.3%) reported macrolide antibiotic use and 1 patient (6.7% [95% CI, 0.3%, 34.0%]) reacted to subsequent treatment.

**Conclusions:** Graded oral challenges can be used to safely diagnose macrolide antibiotic allergy. The risk of subsequent reaction among those with a negative challenge is almost 7%. Guidelines establishing the diagnosis of macrolide allergy are required to appropriately manage these patients.

### A65 Cytokine profile of Th1 and Th2 cells following T cell polarization in high and low atopic risk umbilical cord blood mononuclear cell samples

#### Mallory Gallant^1,2^, Mark W. Tenn^1,2^, Jenny Thiele^1,2^, and Anne K. Ellis^1,2^

##### ^1^Departments of Medicine and Biomedical & Molecular Sciences, Queen’s University, Kingston, ON, Canada; ^2^Allergy Research Unit, Kingston General Hospital, Kingston, ON, Canada

###### **Correspondence:** Mallory Gallant

*Allergy, Asthma & Clinical Immunology* 2018, **14(Suppl 1):**A65

**Background:** Individuals with atopic disorders tend to have a decreased ratio of Th1 to Th2 cells, known as a “Th2 shift”. The dynamic balance between Th1 and Th2 cells in combination with their functionality in early life has not been thoroughly investigated, and may serve as an important predictor of atopic disease. We investigated this prospect by examining cytokine production of newly polarized Th1 and Th2 cells from umbilical cord blood mononuclear cell (CBMC) samples from mothers with and without self-reported allergic disease.

**Methods:** CBMC samples were collected and cryo-preserved from consenting mothers with self-reported allergy (high atopic risk (HR) n = 4) or no allergy (low atopic risk (LR) n = 3). Non-adherent mononuclear cells were isolated from thawed samples and cultured for 7 days under Th1 (Interleukin (IL) 12, IL-2, and anti-IL4), Th2 (IL-4, IL-2, and anti-IL-12), and non-polarizing (IL-2, anti-IL-4 and anti-IL-12) conditions. Culture supernatants were extracted and cytokine concentrations were quantified via Bio-Plex Pro™ Human Cytokine 17-plex Assay.

**Results:** IL-1β, IL-7, IL-8, IL-12, IL-17, IFN-γ, and TNF-α were significantly increased in Th1 cultures when compared to the Th2 polarizing condition (all p < 0.05). Significantly elevated IL-5 and IL-13 levels were observed in Th2 supernatants versus Th1 supernatants (all p < 0.05). HR participants generated numerically higher levels of IL-8 and IL-10 (p = 0.58 and p = 0.21 respectively), and decreased levels of IFN-γ (p = 0.19) when compared to LR counterparts.

**Conclusion:** The polarization culture conditions induced skewing towards a Th1 or Th2 cell phenotype with respect to cytokine concentrations. Given the small sample size of HR and LR participants, only weak trends for cytokine secretion were observed. Experiments with additional participants will improve power and confirm reproducibility of results in order to further address the potential of cytokine profiling in early life as a predictor of atopic disease.

### A66 IVIG for recurrent pregnancy loss and recurrent implantation failure: experience at the MUHC

#### Geneviève Genest^1^, Carl A. Laskin^2^, Phil Gold^1^

##### ^1^Department of Allergy-Immunology, McGill University, Montreal, Quebec, Canada; ^2^Department of Medicine and Obstetrics, University of Toronto, Toronto, Ontario, Canada

###### **Correspondence:** Geneviève Genest

*Allergy, Asthma & Clinical Immunology* 2018, **14(Suppl 1):**A66

**Background:** IVIg is a potential therapy for immune-mediated recurrent pregnancy loss (RPL) and implantation failure (RIF). Due to study heterogeneity and lack of biomarkers to diagnose immune mediated reproductive failure (IMRF), IVIg remains controversial. To take advantage of IVIg’s immunomodulatory potential, we believe that it must be given at sufficient doses before implantation. We have developed a standardized protocol for patients with suspected IMRF.

**Methods:** Single center pilot study conducted at McGill’s Immunology Clinic. We included patients 18–42 with > 4 miscarriages or > 3 failed embryo transfers (ET). IVIg (or equivalent doses of scIg)) 600–800 mg/kg was administered 5–10 days before embryo transfer. If successful pregnancy ensued, IVIg was given monthly until 32 weeks. We prospectively followed patients during pregnancy and 1 month after delivery to determine the efficacy and safety of IVIg.

**Results:** 15 patients were included. 13/15 (86%) have delivered healthy offspring; 2/15 (13%) failed their ET. In the subgroup analysis, successful outcomes occurred in 7/9 (77%) patients with primary RIF, 2/2 (100%) with secondary RIF, 3/3 (100%) with primary PRL and 1/1 (100%) with secondary RPL. IVIg related side effects included 6/15 (40%) reports of mild post-infusion headaches; one patient developed hypotension and chills during her first IVIg infusion but tolerated scIg. IVIg was discontinued prematurely in 2 patients, one because of hospitalization for renal colic and another developed prolonged hypotension at needle insertion. Obstetrical complications were reported in 2 patients, one delivered prematurely at 34 6/7 weeks because of HELLP syndrome, another developed preterm labour at 27 weeks but delivered a healthy child at term. There was one fetal anomaly reported; one child was born with mild benign hydrocephalus.

**Conclusions:** Our IVIg protocol appears to be safe and may be effective for patients with suspected IMRF undergoing IVF and our results justify the need for a properly designed RCT.

### A67 Canadian Immunodeficiency Patients Organization (CIPO) survey of Canadian patients with primary immunodeficiency disease

#### Whitney Goulstone, Erin Tough

##### Canadian Immunodeficiencies Patient Organization (CIPO), Hasting, Ontario, K0L 1Y0, Canada

###### **Correspondence:** Whitney Goulstone

*Allergy, Asthma & Clinical Immunology* 2018, **14(Suppl 1):**A67

**Background:** Primary immunodeficiency diseases (PIDs) represent a significant collection of immune system disorders that increase susceptibility to infection, which in some cases are serious or life-threatening. Patients with PID often require immunoglobulin G (IgG, commonly referred to as Ig) replacement therapy to prevent infections and associated comorbidities. PID treatment, in addition to symptoms and associated social and emotional impacts, has a significant impact on patients’ quality of life (QoL).

**Methods:** The authors conducted a cross-sectional survey to measure health-related QoL in a cohort of patients with PID. Eligible participants were identified through the Canadian Immunodeficiencies Patient Organization (CIPO). The questionnaire consisted of 61 questions that covered patient-reported outcomes including diagnosis, QoL, treatment regimes, and communication.

**Results:** Surveys were returned by 149 patients with PID. Participants conveyed significant impact on QoL including personal, occupational, financial, emotional, and social impacts as a result of PID symptoms, risks, and treatment logistics, limitations, and side effects. The most common diagnoses were related to B-Lymphocyte disorders (60.4%). Common treatments include intravenous immunoglobulin (IVIg; in hospital) and subcutaneous immunoglobulin (SCIg; at home), in addition to antibiotics and antifungals as required. Respondents reported feeling “average” before treatment on a scale of 0–10 (mean 5.46 ± 2.23) with increased health after treatment (mean 6.66 ± 2.32). Respondents felt current treatment was convenient (mean 8.22 ± 1.94) and were comfortable with self-infusions (mean 6.96 ± 3.46).

**Conclusions:** Patients with PID are not uncommon in the Canadian community, and in these patients PID is associated with a significant impairment in QoL. Experiences range with regards to a particular treatment’s advantages and disadvantages, cost, travel, and convenience. Respondents hope to achieve improved QoL through the following solutions: better treatment, improved infusions, gene modification, more research and clinical trials, a cure, and education and outreach. Improved financial, medical, and social supports were also requested.

### A69 Predictors of prognosis for chronic idiopathic urticaria

#### Christina M. Huang^1^, Wilma M. Hopman^2^, and Rozita Borici-Mazi^3^

##### ^1^School of Medicine, Queen’s University, Kingston, Ontario, Canada; ^2^Clinical Research Centre, Kingston Health Sciences Centre; Department of Public Health Sciences, Queen’s University, Kingston, Ontario, Canada; ^3^Division of Allergy & Immunology, Department of Medicine, Queen’s University, Kingston, Ontario, Canada

###### **Correspondence:** Christina M. Huang

*Allergy, Asthma & Clinical Immunology* 2018, **14(Suppl 1):**A69

**Background:** Chronic idiopathic urticaria (CIU) is characterized by wheals and itching for at least 6 weeks with no identifiable cause. Previous studies have suggested a relationship between female gender, asthma and CIU, but little is known about the predictors of prognosis. The purpose of this study was to determine the predictors of disease outcome for patients with persistent CIU.

**Methods:** This was a retrospective chart review of patients 18 years of age and older with persistent CIU (at least 3 clinic visits) seen in the Outpatient Allergy Clinic at Kingston Health Sciences Centre. Patients exhibiting symptoms of physical urticarias were excluded. Patients’ demographics, clinic visits, disease duration and outcome, comorbidities, angioedema, triggers, allergies, medications used, and results of work up were recorded. A two-level disease outcome was used, where “controlled” was defined as urticaria-free patients, on or off medication. Those who relapsed after a period of remission or had persistent symptoms despite medications were considered “uncontrolled”. Analyses included Chi square tests, independent samples t-tests, and a multivariable logistic regression. This study was approved by Queen’s University Ethics Committee.

**Results:** Of the 98 patients included, 83% (81/98) were females. The average age, duration of disease, and number of clinic visits were 51.18 ± 15.12 years, 45.13 ± 69.9 months, and 4.63 ± 2.61, respectively. There was a trend between age and disease outcome, as prognosis was worse in younger patients (p = 0.077). A greater proportion of males obtained control of their disease (82.4%) as compared to females (64.2%) (p = 0.147). Female patients used significantly more medications then males to control their symptoms (2.77 vs 2.0, p = 0.026). Patients with co-existing asthma experienced significantly worsened outcomes as compared to non-asthmatics (p = 0.04).

**Conclusions:** In adults with persistent CIU, younger female asthmatics were less likely to gain control of disease. Physicians should have a lower threshold for escalating treatment strategies for this patient population.

### A70 Phenotypes, endotypes and biomarkers of hypersensitivity reactions to 16 monoclonal antibodies: Management with desensitization in 104 patients

#### Ghislaine A.C. Isabwe^1,2^, Marlene Garcia-Neuer^1^, Leticia De Las Vecillas Sanchez^1,3^, Donna M. Lynch^1^, Kathleen Marquis^1^, Mariana Castells^1^

##### ^1^Division of Rheumatology, Immunology and Allergy; Department of Medicine, Brigham and Women’s Hospital, Harvard Medical School, Boston, Massachusetts, USA; ^2^Division of Allergy and Immunology; Department of Pediatrics, Centre Hospitalier Universitaire de Sherbrooke, Université de Sherbrooke, Sherbrooke, Quebec, Canada;^3^Department of Allergy, Marqués de Valdecilla University Hospital-IDIVAL, Santander, Spain

###### **Correspondence:** Ghislaine A.C. Isabwe

*Allergy, Asthma & Clinical Immunology* 2018, **14(Suppl 1):**A70

**Background:** Increase in hypersensitivity reactions (HSRs) have been observed with the use of monoclonal antibodys (mAbs) and has prevented the use of first-line therapies. The current classification of HSRs to mAbs do not conform to current clinical presentations. Management of HSR to mAbs includes desensitization which provides a safe and efficient avenue for their re-introduction. Our objective is to propose a new classification for HSRs to mAbs and provide new insight into their management with desensitization.

**Methods:** We reviewed the characteristics of the presentation of initial HSRs for 104 patients, skin testing, biomarkers and the outcomes of their management with 526 desensitizations.

**Results:** The initial reaction phenotypes included type I (IgE/non IgE mast cell activation) in 65 (63%), cytokine-release in 14 (13%), mixed reactions (Type I/cytokine) in 22 (21%) and delayed type IV in 3 (3%) patients. Skin testing was performed for 10 mAbs in 58 patients. A positive skin test was associated with severe initial reaction (p-value 0.0088). IL-6 was elevated in 8 patients, correlated with cytokine storm phenotype. Tryptase was elevated in 1 patient. There were no HSR during 404 (76.8%) desensitizations; when reactions occured, they were type I in 39 (32%), cytokine storm in 64 (52.5%), mixed in 14 (11.5%) and delayed type IV in 5 (4%). All but one patient safely completed their desensitization and received their target dose.

**Conclusions:** A new proposed classification for HSRs to mAbs is provided based on the presentation and includes 4 phenotypes; type I (IgE/non-IgE mast cells activation), cytokine storm, mixed (type I/cytokine release) and delayed type IV reactions. Endotypes were confirmed by tryptase and IL-6. Skin testing is a valuable tool in assessing HSRs to mAbs. Management with desensitization provides safe and effective re-treatment options. Precision and personalized medicine should be applied to patients with HSR to mAbs.

### A71 The impact of IgE-mediated activation on human mast cell responses to viral infection

#### Christopher R. Liwski, Liliana Portales-Cervantes, Jean S.Marshall

##### Microbiology and Immunology, Dalhousie University, Halifax, Nova Scotia, B3H 1X5, Canada

###### **Correspondence:** Christopher R. Liwski

*Allergy, Asthma & Clinical Immunology* 2018, **14(Suppl 1):**A71

**Background**: Mast cells play a key role in asthma. The release of preformed mediators, generation of lipid mediators, and production of cytokines, such as GM-CSF, contribute to inflammation and bronchoconstriction. Asthma exacerbations are often associated with respiratory viral infections. Severe asthma is also associated with reduced production of type I and III interferons (IFNs). Human mast cells have been described as an excellent source of IFNs [1]. Therefore, this study investigated the impact of concurrent FcεRI-mediated activation and viral infection on mediator production by human cord blood-derived mast cells (CBMCs).

**Methods**: CBMCs were sensitized with IL-4 (10 ng/mL) and IgE (2.5 µg/mL), and then activated with 2 µg/mL anti-IgE (n = 3). In some experiments, sensitized CBMCs were infected with reovirus (5 MOI) prior to activation with anti-IgE (n = 2). FcεRI expression was measured by flow cytometry. Degranulation was assessed via a β-hexosaminidase assay. IFNA2, IFNL1 and GM-CSF mRNA expression were assessed by qPCR and normalized to the reference gene HPRT.

**Results**: IgE-sensitized CBMCs exhibited increased FcεRI expression (51.5%) compared to unsensitized controls (0.2%), and activation with anti-IgE induced degranulation at 30 min (34.2% vs 10.5% in controls). GM-CSF mRNA expression at 24 h was enhanced 7.122-fold by FcεRI activation, 12.67-fold by reovirus infection, and 55.15-fold by concurrent activation of these pathways compared to non-activated controls. IFNA2 and IFNL1 expression was also induced by reovirus infection. However, substantial donor variation in IFN responses to combined viral and IgE-mediated activation was observed.

**Conclusion:** Our preliminary data suggests that reovirus infection of IgE-activated human mast cells synergistically enhances FcεRI-induced GM-CSF gene expression, but may have a more donor-dependent effect on viral-induced IFN production.

### A72 Natural history, long term outcomes, and treatment response for patients with idiopathic angioedema

#### Amin Kanani, Hanan Ahmed, Raymond Mak

##### Division of Allergy and Immunology, University of British Columbia, Vancouver, British Columbia, Canada

###### **Correspondence:** Raymond Mak

*Allergy, Asthma & Clinical Immunology* 2018, **14(Suppl 1):**A72

**Background:** Idiopathic angioedema (IA) is a form of angioedema in which no known cause can be found. Phenotypically it is divided into histaminergic angioedema which responds to antihistamines, whereas non-histaminergic does not. Studies on the natural history and long term outcomes of idiopathic angioedema are lacking.

**Methods:** Charts of patients with angioedema from an allergist office were reviewed. Patients over age 19 with an initial presentation of IA were recruited to the study. Telephone surveys were conducted to obtain data including demographics, disease characteristics and treatment.

**Results:** 29 patients participated in the survey. Median age of disease onset was 45 years.

Most patients reported attacks lasting less than 24 h. Body parts affected include lips (72%), eyelids (69%), tongue (66%), other facial structures (59%), extremities (41%), airways (38%) and trunk (21%). 59% feel attacks have become less frequent, 45% less severe and 31% shorter over time. 2 patients developed urticaria.

24% of patients report having had a life threatening event. One required intubation. 66% of patients presented at least once to the ER. Antihistamines are the most commonly prescribed treatment in the ER (79%). 53% received epinephrine. 84% responded to treatment.

34% are taking prophylactic antihistamines and 41% take them as needed. 74% of patients find antihistamines effective. 7/29 patients have not had an attack in the last 12 months and do not currently take antihistamines.

Patients report stress, viral illnesses, alcohol, NSAIDS, pressure, insect bites and food additives as potential triggers.

**Conclusions:** Idiopathic angioedema is a chronic illness with variability in frequency and duration of attacks. Angioedema may affect any body part but most commonly affects facial structures. Only a minority of patients will enter remission (no attacks in 12 months) but most patients improve symptomatically over time. Patients perceive antihistamines to be effective prophylactically and as needed.

### A73 Equivalence evaluation of valved holding chambers (VHCs) with albuterol pressurized metered dose inhaler (pMDI)

#### Mark Nagel^1^, Jason Suggett^1^, Vladimir Kushnarev^1^, Andrew McIvor^2^

##### ^1^Trudell Medical International, London, Ontario, Canada; ^2^Firestone Institute of Respiratory Health, St. Joseph’s Healthcare, Hamilton, Ontario, Canada

###### **Correspondence:** Mark Nagel

*Allergy, Asthma & Clinical Immunology* 2018, **14(Suppl 1):**A73

**Background:** VHCs are medical devices that are recommended by Asthma and COPD guidelines to be used with pMDI as a delivery system. VHCs improve medication delivery and reduce oropharyngeal deposition of medication; this is reflected in the fine particles mass (FPM, 1.1–4.7 µm) that reaches the airways of the lungs. The significant role of the VHC in drug delivery was acknowledged by the European Medicines Agency (EMA) in their recommendation that pMDI manufacturers should nominate at least 1 named VHC during clinical safety and efficacy studies. It also recommends if a VHC is to be substituted by an alternative VHC, that appropriate in vitro methods be employed to demonstrate equivalence.

**Methods:** Particle size measurements from the Inspirachamber, Optichamber Diamond, Space chamber, Vortex and Life Brand VHCs (test devices) were compared to the AeroChamber Plus* Flow-Vu* Antistatic VHC (reference device) and made with an Andersen 8-stage cascade impactor operated at 28.3 L/min with Ventolin^®^-HFA pMDIs. The EMA guideline requires comparisons to be performed by justified groupings of stages and recommends at least 4 groups based on physiological relevance. Since a traditional t-test is inappropriate to demonstrate true equivalence a two-one-sided test (TOST) was used.

**Results:** The values for each of the 5 test devices at each of the 4 particle size groupings were outside acceptance criteria for equivalence, thus clearly demonstrating non-equivalence to the reference device.

**Conclusions:** The drug delivery performance from AeroChamber Plus* Flow-Vu* AVHC was significantly different to all test VHCs, none of which passed a test for equivalence. Interchanging of such VHCs with the reference VHC may therefore result in safety and/or efficacy implications unless otherwise proven via in vivo studies.

### A74 Omalizumab treatment response after dose step-up in patients with chronic diopathic/spontaneous urticaria (CIU/CSU): results from the OPTIMA study

#### Wayne Gulliver^1^, Gordon Sussman^2^, Jacques Hébert^3^, Charles W. Lynde^4^, Kim A. Papp^5^, William H. Yang^6^, Olivier Chambenoit^7^, Antonio Vieira^8^, Frederica DeTakacsy^8^, Lenka Rihakova^8^

##### ^1^Faculty of Medicine, Memorial University of Newfoundland, St. John’s, Newfoundland, Canada; ^2^Department of Medicine, University of Toronto, Toronto, Ontario, Canada; ^3^Department of Medicine, Centre Hospitalier de l’Université Laval, Québec, Quebec, Canada; ^4^Lynde Institute for Dermatology, Markham, Ontario, Canada; ^5^K. Papp Clinical Research, Waterloo, Ontario, Canada; ^6^Ottawa Allergy Research Corporation, University of Ottawa Medical School, Ottawa, Ontario, Canada; ^7^Novartis Pharmaceuticals Corporation, East Hanover, New Jersey, USA; ^8^Novartis Pharmaceuticals Canada Inc., Dorval, Quebec, Canada

###### **Correspondence:** Lenka Rihakova

*Allergy, Asthma & Clinical Immunology* 2018, **14(Suppl 1):**A74

**Background:** A key secondary objective of the Phase 3b, randomized, open-label, non-comparator OPTIMA study (NCT02161562) was to evaluate omalizumab response in patients with CIU/CSU who step up therapy from 150 to 300 mg.

**Methods:** Patients with CIU/CSU and symptomatic despite H1-antagonists were randomized 4:3 to omalizumab 150 or 300 mg for 24 weeks (1st dosing period). All well-controlled patients (UAS7 ≤ 6) were then subjected to treatment withdrawal for up to 8 weeks. The patients whose symptoms came back (UAS7 ≥ 16) within this timeframe were retreated at the same dose. The patients who did not achieve remission during the 1st dosing period were either: (1) stepped-up (150–300 mg) if symptoms were not controlled after ≥ 8 and ≤ 24 weeks; or (2) had treatment extension if symptoms were not well-controlled with 300 mg at 24 weeks.

**Results:** A total of 314 patients (73% female, 79% white, mean age 46 years, mean baseline UAS7 score 29.8) were randomized to either 150 mg (n = 178) or 300 mg (n = 136) omalizumab. After initial treatment, 64.7% treated with 300 mg were well-controlled (UAS7 ≤ 6). In the 150 mg arm, 27 (15.2%) were well-controlled (UAS7 ≤ 6) and 141 stepped-up to 300 mg between week 8–24 as their symptoms were not controlled (UAS7 > 6). Most patients (115/141; 81.5%) up-dosed after 2.150 mg omalizumab doses (8 weeks), and the remaining 26 lost symptom control (UAS7 > 6) and up-dosed later during the initial dosing. One hundred and thirty (130) of the stepped-up patients completed the 3-dose step-up period. Of these, 59/130 (45.4%) patients achieved symptom control (UAS7 ≤ 6) and 33/130 (25.4%) had complete response (UAS7 = 0). In contrast, 55.9% of patients initially randomized to 300 mg achieved UAS7 ≤ 6 after three doses.

**Conclusions:** Most CIU/CSU patients treated with 150 mg omalizumab had to up-dose to 300 mg because of insufficient symptom control. About half of up-dosed patients achieved symptom control following 3 doses of 300 mg omalizumab.

### A75 Omalizumab retreatment of patients with chronic idiopathic/spontaneous urticaria (CIU/CSU) after initial response and relapse: primary results of the OPTIMA Study

#### Gordon Sussman^1^, Jacques Hébert^2^, Wayne Gulliver^3^, Charles Lynde^4^, William H. Yang^5^, Olivier Chambenoit^6^, Antonio Vieira^7^, Frederica DeTakacsy^7^ and Lenka Rihakova^7^

##### ^1^Department of Medicine, University of Toronto, Toronto, Ontario, Canada; ^2^Department of Medicine, Centre Hospitalier de l’Université Laval, Québec, Quebec, Canada; ^3^Faculty of Medicine, Memorial University of Newfoundland, St. John’s, Newfoundland, Canada; ^4^Lynde Institute for Dermatology, Markham, Ontario, Canada; ^5^Ottawa Allergy Research Corporation, University of Ottawa Medical School, Ottawa, Ontario, Canada; ^6^Novartis Pharmaceuticals Corporation, East Hanover, New Jersey, USA; ^7^Novartis Pharmaceuticals Canada Inc., Dorval, Quebec, Canada

###### **Correspondence:** Lenka Rihakova

*Allergy, Asthma & Clinical Immunology* 2018, **14(Suppl 1):**A75

**Background:** The primary objective of the Phase 3b, randomized, open-label, non-comparator OPTIMA study (NCT02161562) was to assess omalizumab retreatment of patients with CIU/CSU.

**Methods:** Patients with CIU/CSU and symptomatic despite H1-antagonists were randomized 4:3 to Omalizumab 150 or 300 mg for 24 weeks (1st dosing period). All well-controlled patients (UAS7 ≤ 6) were then subjected to treatment withdrawal for up to 8 weeks: Patients whose symptoms came back (UAS7 ≥ 16) within this timeframe were retreated at the same dose as in the 1st dosing period. The patients who did not achieve remission during the 1st dosing period were either: (1) stepped-up (150–300 mg) if symptoms were not controlled after ≥ 8 and ≤ 24 weeks; or (2) had treatment extension if symptoms were not well-controlled with 300 mg at 24 weeks.

**Results:** There were 314 patients (73% female, 79% white, mean age 46 years, mean baseline UAS7 score 29.8) randomized to either 150 mg (n = 178) or 300 mg (n = 136) Omalizumab. After 1st dosing period, 15.2% (150 mg dose) and 64.7% (300 mg dose) of patients were well-controlled. After withdrawal, 44% of patients on 150 mg and 50% on 300 mg relapsed within 8 weeks. Mean time to relapse was 4.8 (150 mg) and 4.7 (300 mg) weeks. Upon retreatment, most patients achieved UAS7 ≤ 6 (150 mg: 83.3% [95% CI, 62.2 − 100%]; 300 mg: 89.2% [95% CI, 79.2 − 99.2%]). In responders, mean time to response was similar between the 1st and 2nd dosing periods (3.5 vs 3.1 weeks). Of all retreated patients (n = 56), 80% (1st period) and 85% (2nd period) achieved symptom control (UAS7 ≤ 6) and 63% (1st period) and 56% (2nd period) achieved complete response (UAS7 = 0) after two doses. Omalizumab was well-tolerated throughout.

**Conclusions:** Omalizumab retreatment is safe and effective in patients with CIU/CSU who respond to initial treatment and later relapse; most patients regain symptom control after a 2nd course.

### A76 Design and rationale of OPTIMA, a study to evaluate retreatment, extension, or step-up therapy with omalizumab in patients with chronic idiopathic/spontaneous urticaria (CIU/CSU)

#### Gordon Sussman^1^, Jacques Hébert^2^, Wayne Gulliver^3^, Charles Lynde^4^, William H. Yang^5^, Olivier Chambenoit^6^, Gretty Deutsch, Frederica DeTakacsy^7^, Lenka Rihakova^7^

##### ^1^Department of Medicine, University of Toronto, Toronto, Ontario, Canada; ^2^Department of Medicine, Centre Hospitalier de l’Université Laval, Québec, Quebec, Canada; ^3^Faculty of Medicine, Memorial University of Newfoundland, St. John’s, Newfoundland, Canada; ^4^Lynde Institute for Dermatology, Markham, Ontario, Canada; ^5^Ottawa Allergy Research Corporation, University of Ottawa Medical School, Ottawa, Ontario, Canada; ^6^Novartis Pharmaceuticals Corporation, East Hanover, New Jersey, USA; ^7^Novartis Pharmaceuticals Canada Inc., Dorval, Quebec, Canada

###### **Correspondence:** Lenka Rihakova

*Allergy, Asthma & Clinical Immunology* 2018, **14(Suppl 1):**A76

**Background:** In Phase 3 studies, subcutaneous Omalizumab (150 or 300 mg every 4 weeks for 24 weeks) was safe and effective in treating symptoms associated with CIU/CSU. OPTIMA (NCT02161562) is a novel study addressing remaining gaps in knowledge of optimal CIU/CSU treatment.

**Design:** Patients with CIU/CSU and symptomatic despite H1-antagonists were randomized 4:3 to Omalizumab 150 or 300 mg for 24 weeks (1st dosing period). All well-controlled patients (UAS7 ≤ 6) were then subjected to treatment withdrawal for up to 8 weeks. The patients whose symptoms came back (UAS7 ≥ 16) within this timeframe were retreated at the same dose as in the 1st dosing period. The patients who did not achieve remission during the 1st dosing period were either (1) stepped-up (150–300 mg) if symptoms were not controlled after ≥ 8 and ≤ 24 weeks; or (2) had treatment extension if symptoms were not well-controlled with 300 mg at 24 weeks. The entire study was 53 weeks. To observe a sufficient number of relapses after initial dosing with 150 or 300 mg, 314 patients were enrolled.

**Analysis:** The primary endpoint was the proportion of patients who were clinically well controlled (UAS7 ≤ 6) after the initial dosing phase, relapsed (UAS7 ≥ 16) upon withdrawal, and who achieved a UAS7 score ≤ 6 at the end of the second dosing phase. Key secondary endpoints include: change in UAS7 score and proportion of patients UAS7 ≤ 6 in those who step-up from 150 to 300 mg; change in UAS7 score in patients who extend 300 mg treatment; time to relapse in both doses.

**Conclusions:** This study helps identify appropriate Omalizumab treatment in CIU/CSU patients who relapse or are not well controlled after initial treatment.

### A77 Assessing the validity and reliability of a penicillin allergy de-labeling questionnaire in pediatric patients

#### Hannah Roberts^1^, Christopher Mill^1^, Karen Ng^2^, Edmond S. Chan^1^, Kyla Hildebrand^1^, Tiffany Wong^1^

##### ^1^Department of Pediatrics, University of British Columbia, Vancouver, British Columbia, Canada; ^2^Faculty of Pharmaceutical Sciences, University of British Columbia, Vancouver, British Columbia, Canada

###### **Correspondence:** Hannah Roberts

*Allergy, Asthma & Clinical Immunology* 2018, **14(Suppl 1):**A77

**Background:** Among patients who report a penicillin allergy, more than 80% have negative testing. Patients can be erroneously labeled with a penicillin allergy due to a misclassification of the suspected reaction. This study seeks to validate a questionnaire and assessment tool that will guide physicians in identifying penicillin allergy risk groups among pediatric patients.

**Methods:** The questionnaire was developed by pediatric allergists to assess history of possible penicillin allergy. Subjects are recruited from referrals to the allergy clinic for penicillin allergy assessment. Pharmacists and allergists administer the questionnaire to participants during the visit. The questionnaire answers will be assessed for inter-rater reliability. The allergist assessment and outcome from the clinic visit will be compared with follow up clinical assessment and decision forms (completed by allergy and non-allergy physicians) to assess validity.

**Results:** We report the results of a preliminary analysis from the first 24 patients recruited between November 2016 and March 2017. 46% were male and the median age was 7 years. 79% received amoxicillin. 71% subjects reported a maculopapular rash, 42% of subjects reported urticaria. Symptoms lasted > 48 h in 55% of cases. 96% of subjects had consulted medical advice. Skin testing was not indicated in 66%. 19 subjects received amoxicillin oral challenge, and none reacted. Of 24 subjects assessed, 22 (91%) were found not to be allergic, one was deemed allergic to penicillin and one was diagnosed with severe adverse drug reaction.

**Conclusions:** Most patients received amoxicillin and presented with prolonged maculopapular rashes or urticaria. The majority of subjects referred were deemed not allergic after an allergist assessment. Most patients are not deemed allergic based on history alone and pass drug challenges, without need for skin testing. The availability of a clinical tool to guide physicians in assessing risk level for possible penicillin allergy would decrease risk of erroneous penicillin allergy labels.

## Case Reports

### A85 Systemic strawberry reaction in a 6-month-old with negative ImmunoCAP and skin tests

#### Adil Adatia^1^, Thomas Gerstner^2^, Diane Marks^2^, Tamar Rubin^2^

##### ^1^Department of Internal Medicine, University of Alberta, Edmonton, Alberta, Canada; ^2^Department of Pediatrics and Child Health, University of Manitoba, Winnipeg, Manitoba, Canada

###### **Correspondence:** Adil Adatia

*Allergy, Asthma & Clinical Immunology* 2018, **14(Suppl 1):**A85

**Background:** Although adverse strawberry reactions are anecdotally common in children, there are few published reports and no published cases of strawberry anaphylaxis in infants. We describe an infant with anaphylactic reactions to strawberry, but negative skin and ImmunoCAP tests.

**Case presentation:** A previously healthy, otherwise non-atopic 6-month-old male was referred for evaluation after two suspected strawberry reactions. At age 5.5 months, he developed urticaria immediately after his first known ingestion of strawberry. At age 6 months he developed physician-documented urticaria, and parent-reported emesis and stridor after ingesting fresh strawberry. Four weeks later, skin prick testing (SPT) with strawberry extract and prick-to-prick testing with fresh strawberry and other rosaceae fruit was negative. A graded open challenge to fresh strawberry resulted in projectile emesis, cough, stridor, auscultated wheeze, and mottling (without angioedema or urticaria) 5 min after his second dose (0.6 g). He received two doses of epinephrine, salbutamol and desloratadine. ImmunoCAP was negative to several rosaceae fruits, including strawberry and raspberry. Six weeks later, repeat SPT with fresh strawberry puree resulted in a flare-only reaction on his back, but not his forearm. SPT with peach skin, peach flesh and birch was negative. Serum tryptase is pending.

**Discussion:** Known strawberry allergens include the Bet v 1 homologue Fra a 1 (implicated in pollen-food allergy syndrome), the profilin Fra a 4, and the lipid transfer protein (LTP) Fra a 3. Cases of isolated urticaria and angioedema in children due to strawberry are generally attributed to non-immunologic histamine release induced by a strawberry glycoprotein. Our inability to detect strawberry sensitization in our patient suggests an exaggerated form of non-specific histamine release. However, given his anaphylactic presentation and theoretical risk for co-allergy with other rosaceae fruits, it is essential to rule out LTP sensitization.

**Consent:** Written consent was obtained from the patient’s mother.

### A86 Hepatopulmonary syndrome secondary to possible nodular regenerative hyperplasia in a patient with common variable immunodeficiency

#### Adil Adatia^1^, Chrystyna Kalicinsky^2^

##### ^1^Department of Internal Medicine, University of Alberta, Edmonton, Alberta, Canada; ^2^Department of Internal Medicine, Section of Clinical Immunology and Allergy, University of Manitoba, Winnipeg, Manitoba, Canada

###### **Correspondence:** Adil Adatia

*Allergy, Asthma & Clinical Immunology* 2018, **14(Suppl 1):**A86

**Background:** Common variable immunodeficiency (CVID) is associated with numerous non-infectious manifestations, including chronic liver disease. Nodular regenerative hyperplasia (NRH) has been identified as the most common form of liver pathology in CVID patients, with these patients being at risk of the attendant complications of hepatic dysfunction. We present a case of hepatopulmonary syndrome in a CVID patient with possible NRH.

**Case presentation:** A 52-year-old male with CVID on immunoglobulin replacement for approximately 30 years presented with progressive dyspnea out of keeping of his known mild bronchiectasis. He had previously been investigated for liver disease based on elevated liver enzymes, splenomegaly, and thrombocytopenia; however, the liver biopsy showed only non-specific sinusoidal fibrosis without evidence of cirrhosis. Pulmonary function testing was notable for a DLCO of 50% predicted and an alveolar-arterial gradient of 50 mmHg. Lung perfusion imaging was positive for a right-to-left shunt and a contrast-enhanced transthoracic echocardiogram confirmed extracardiac shunting. There was also evidence of portal hypertension with esophageal varices and portal hypertensive gastropathy. Repeat liver biopsy showed sinusoidal dilatation and fibrosis with features of NRH but was non-diagnostic.

**Discussion:** This case illustrates the possibility of complex, multiorgan dysfunction in CVID. A diagnosis of hepatopulmonary syndrome is made by demonstrating impaired oxygenation and intrapulmonary vascular dilatations in a patient with liver disease, and it should be considered in the evaluation of dyspnea in CVID patients when more common causes have been excluded. Liver transplant, the only treatment for hepatopulmonary syndrome, should not be withheld from CVID patients on the basis of their immunodeficiency given that long-term survival in CVID transplant recipients has been documented.

**Consent:** Written consent was obtained from the patient.

### A87 Anaphylactic reactions to blood products: case series

#### Zeyana Al-Hadhrami^1^, Rozita Borici-Mazi^2^

##### ^1^Department of Medicine, Queen’s University, Kingston, Ontario, Canada; ^2^Division of Allergy & Immunology, Department of Medicine, Queen’s University, Kingston, Ontario, Canada

###### **Correspondence:** Zeyana Al-Hadhrami

*Allergy, Asthma & Clinical Immunology* 2018, **14(Suppl 1):**A87

**Background:** Anaphylactic reaction is a rare complication of transfusion of blood products. Platelet components provide the highest rate of allergic reactions, followed by fresh frozen plasma (FFP). Methylene blue has been considered the culprit in few European case series, however, there is little information on the cause of allergic reactions to blood products in North America. We described 4 cases of confirmed anaphylactic reactions to blood products.

**Methods:** We reviewed literature from OvidSP and PubMed using search terms allergy and fresh frozen plasma, allergy and blood products. All four patients were evaluated in the Outpatient Allergy Clinic at Kingston Health Sciences Centre. Detailed history, including the sequence of events during the anaphylactic reaction, past medical and drug history, previous exposure to blood products, and self and family history of drug allergy or atopy was obtained. Skin prick tests followed by intradermal testing to FFP and Human Serum Albumin (HAS) were performed. Serum IgA levels were measured.

**Results:** Ninety-five articles were identified and nine articles were selected for complete review. Four patients (3 males and 1 female) were investigated for possible allergy to blood products. Three patients experienced symptoms of anaphylaxis after administration of FFP and one following HAS. All patients had previous exposures to FFP. Patient who reacted to HAS did not have previous exposure to any blood products. All patients had positive skin testing to either FFP or HSA via prick or intradermal method. IgA deficiency was ruled out in three out four patients. All patients were provided with adequate education regarding future transfusions as well as a medical alert bracelet.

**Conclusion:** Anaphylactic reactions to blood products require work up by allergist and close collaboration with Blood Transfusion Services. Future studies are required to investigate the possible cause(s) of allergic reactions to blood products in North America.

### A88 The safety of etoposide phosphate administration in pediatric patients with an etoposide hypersensitivity

#### Joel P. Brooks, Darren Luon, Alexandra Helgeson, Hannah Martin, Christina Price

##### Department of Allergy and Immunology, Yale University School of Medicine, New Haven, Connecticut, United States of America

###### **Correspondence:** Joel P. Brooks

*Allergy, Asthma & Clinical Immunology* 2018, **14(Suppl 1):**A88

**Background:** Podophyllotoxin etoposide is a chemotherapeutic agent in use for over 30 years. Type I hypersensitivity reactions occur in 1–3% of patients receiving etoposide. We discuss two pediatric patients with etoposide hypersensitivities successfully treated with etoposide phosphate without the need for desensitization.

**Case study:** Patient 1: A 14 year-old male with relapsed osteosarcoma status post right leg above the knee amputation and left thoracotomy with left lung wedge resection was initially treated with a chemotherapy regimen containing high dose methotrexate, cisplatin, and doxorubicin from 2012 to 2013. In 2016, a relapse in his lung was identified and he was started on ifosfamide and etoposide. Within 1 min of receiving his first etoposide dose, he developed respiratory distress and lip swelling. His infusion was stopped, diphenhydramine was administered, and vitals remained stable. The patient was started on etoposide phosphate and completed six cycles of therapy without any adverse reactions. Patient 2: A 13 year-old male with metastatic Ewing’s sarcoma was treated with vincristine, doxorubicin, mesna, and cyclophosphamide, alternating with cycles of ifosfamide, mesna, and etoposide. Within 1 min of his first etoposide dose, he developed a cough, generalized pallor, facial edema, and had a large emesis. His oxygen saturation decreased to the 80 s and blood pressure dropped to 70 s systolic. Diphenhydramine and a saline bolus were administered with stabilization of his vitals and symptom resolution. The patient was started on etoposide phosphate and tolerated 14 cycles.

**Discussion:** The mechanism by which etoposide causes a hypersensitivity reaction is suspected to be caused by the polysorbate 80 component. Neither oral etoposide, nor etoposide phosphate contain polysorbate 80 and are usually safe for administration. However, caution is needed as there are cases of etoposide phosphate hypersensitivity.

**Conclusion:** Etoposide phosphate is a potentially safe alternative for pediatric patients with etoposide hypersensitivity.

**Consent:** Informed consent has been obtained from the patients and next of kin reported in this abstract.

### A90 Adverse cutaneous reaction to subcutaneous immunoglobulin therapy in patients with immunodeficiency

#### Irene I. Chair^1^, Donald F. Stark^2^

##### ^1^Department of Medicine, University of British Columbia, Vancouver, British Columbia, Canada; ^2^Division of Allergy and Immunology, Department of Medicine, University of British Columbia, Vancouver, British Columbia, Canada

###### **Correspondence:** Irene I. Chair

*Allergy, Asthma & Clinical Immunology* 2018, **14(Suppl 1):**A90

**Case study:** Treatment with human immunoglobulin is indicated in various medical conditions including autoimmune diseases, infections and immunodeficiency syndromes. Serious cutaneous adverse reactions due to immunoglobulin therapy are rare and most often associated with patients with neurologic conditions who are receiving higher doses of intravenous immunoglobulin (IVIG) infusions. We report two cases of rash associated with patients receiving subcutaneous immunoglobulin (SCIG) therapy for IgG deficiency. One patient developed a painful erythematous inframammary rash soon after the initiation of SCIG therapy, persisting beyond the cessation of therapy. Trial of a different SCIG product also resulted in similar rash. The second patient developed a blistering erythematous rash on the legs after a few years of uneventful immunoglobulin therapy. The mechanism of these cutaneous reactions is not clear. There are a number of proposed theories in the literature for rash associated with immunoglobulin therapy, such as T cell mediated pathway, complement mediated immune complex deposition and IgE involvement. Although these cutaneous reactions were not life threatening, they resulted in the cessation of a beneficial therapy. Further analysis of similar cases may be helpful to understand the underlying pathophysiology of these reactions.

**Consent:** Informed verbal consent to publish is obtained from both patients with written consent to follow.

### A91 First-reported pediatric case of anaphylaxis to American ginseng

#### Stephanie C. Erdle^1^, Edmond S. Chan^2^, Hyungjun Yang^3^, Bruce A. Vallance^3^, Christopher Mill^2^, Tiffany Wong^2^

##### ^1^Division of Pediatric Medicine, Department of Pediatrics, The Hospital for Sick Children, Toronto, Canada; ^2^Division of Allergy & Immunology, Department of Pediatrics, British Columbia Children’s Hospital, Vancouver, Canada; ^3^Division of Gastroenterology, Department of Pediatrics, British Columbia Children’s Hospital, Vancouver, Canada

###### **Correspondence:** Stephanie C. Erdle

*Allergy, Asthma & Clinical Immunology* 2018, **14(Suppl 1):**A91

**Background:** Ginseng has become increasingly popular in North America due to its proposed medicinal effects. Although cases of anaphylaxis have been reported in adults in response to Korean ginseng, there are no reported cases of allergy to American ginseng, nor reported cases in pediatric patients. We present a unique case of anaphylaxis to American ginseng in a 6-year-old girl.

**Clinical case:** A 6-year-old girl with a history of multiple IgE-mediated food allergies (peanuts, tree nuts and fish), atopic dermatitis, and a remote history of asthma was presented to the emergency department with urticaria, coughing, and wheezing. Symptoms began minutes after entering a store selling powdered American ginseng. On physical examination, she had wheezing bilaterally and diffuse urticaria. She was treated with salbutamol, dexamethasone and diphenhydramine. Symptoms resolved shortly after treatment, and she was referred for allergy testing.

Skin prick testing (SPT) with American ginseng was positive with a 13x12 mm wheal. Spirometry was entirely normal (FEV1 107% predicted). A basophil activation test (BAT) showed a dose-dependent increase in expression of CD63 on basophils in response to American ginseng extract, but not to Korean ginseng extract. No changes were observed in a non-atopic control, and minimal changes were observed in an atopic control. It was concluded that this patient had an anaphylactic reaction to American ginseng. She was advised to strictly avoid all ginseng products and carry an epinephrine autoinjector at all times.

**Conclusion:** We present a unique case of anaphylaxis with physician-confirmed respiratory symptoms and urticaria, with evidence of sensitization to ginseng on SPT and allergy on BAT. This is the first reported case of ginseng anaphylaxis in a pediatric patient, in addition to anaphylaxis to American ginseng. As ginseng exposure is becoming increasingly prevalent in North America, it is important to consider ginseng as a cause of allergic presentation in children.

**Consent to publish:** Written informed consent to publish was obtained from the patient’s guardians.

### A92 Mepolizumab for severe chronic rhinosinusitis with polyposis: a case report

#### Samira Jeimy^1^, Godfrey Lam^1^, D. William Moote^1^, Brian Rotenberg^2^

##### ^1^Department of Clinical Immunology and Allergy, Western University, London, Ontario, Canada; ^2^Department of Otolaryngology, Western University, London, Ontario, Canada

###### **Correspondence:** Samira Jeimy

*Allergy, Asthma & Clinical Immunology* 2018, **14(Suppl 1):**A92

**Background:** Chronic rhinosinusitis (CRS) is an inflammatory disorder of the nasal passage lining and paranasal sinuses that affects 5–15% of the American population, with significant quality of life and health resource utilization burden. CRS with nasal polyposis (CRSwNP) is a CRS subtype that often requires multiple endoscopic sinus surgeries and frequently associated with asthma and allergic rhinitis. CRSwNP involves TH2 eosinophilic inflammation with high IL-5 and IgE levels. Several clinical trials have demonstrated effectiveness of anti-IL5 monoclonal antibodies (mAb) therapy in the management of eosinophilic disorders.

**Case:** We present a case of a 22-year old security officer with severe CRSwNP and aspirin associated respiratory disease (AERD). His medical history included asthma with ragweed pollen sensitization, elevated serum IgE (122), and peripheral eosinophilia (500/μL). Investigations for vasculitis, aspergillus sensitization, immunodeficiency, cystic fibrosis, and primary ciliary disorders were negative. Despite multiple endoscopic sinus surgeries and maximal medical therapy (saline and 0.5 mg budesonide irrigation, and aspirin 650 mg, twice daily), his symptoms persisted. Mucocele development and invasion into the ipsilateral orbit led to orbital abscess formation and visual compromise, necessitating urgent surgical decompression. Intra-operatively, the middle and superior turbinates were noted to have complete polypoid transformation. Based on his history of refractory CRSwNP, asthma, and peripheral eosinophilia, he was started on the anti-IL5 mAb mepolizumab. After three monthly 100 mg subcutaneous infusions, he had significant improvement in nasal obstruction and purulent rhinorrhea, and complete return of olfaction. His visual acuity and extra-ocular movements returned to baseline. Endoscopy revealed no evidence of polyp recurrence or ongoing inflammation.

**Conclusion:** Several clinical trials have evaluated the safety and efficacy of biologics in CRSwNP. The anti-IgE monoclonal antibody omalizumab and anti-IL-4 receptor antibody dupilumab demonstrated efficacy in reducing polyp size and improving nasal symptoms as long term therapy. Anti-IL-5 therapy has only been studied as single dose treatment. Our case illustrates the potential therapeutic benefit of extended anti-IL-5 therapy to induce disease remission in patients with medically and surgically refractory CRSwNP.

**Consent:** Written informed consent for the publication of these details was obtained from the patient.

### A93 Mast cell disorder associated with adult onset food anaphylaxis: a case report

#### Samira Jeimy, Nazanin Montazeri, Harold Kim

##### Department of Clinical Immunology and Allergy, Western University, London, Ontario, Canada

###### **Correspondence:** Samira Jeimy

*Allergy, Asthma & Clinical Immunology* 2018, **14(Suppl 1):**A93

**Background:** Food allergy affects approximately 2% of adults. The most common adult triggers include fruits and vegetables; delayed sensitization may develop due to shared homologous proteins with airborne allergens (i.e., pollens). Mast cells are associated with allergic diseases, including asthma, atopic dermatitis, and medication hypersensitivity. In contrast, the association of adult onset food allergy with mast cell disorder has not been well described.

**Case study:** We present a case of a 55-year-old man with a three-year history of recurrent syncope, associated with almond and yellow pepper ingestion. He also reported sporadic flushing and pruritus without eruption. Skin testing with fresh extracts of almonds and yellow pepper was positive. Baseline serum tryptase levels were elevated, with serial values of 29, 32, and 37 (normal < 11.4) ng/mL. Investigations included normal serum immunoglobulins, protein electrophoresis, and 24-h urine 5-hydroxyindoleacetic acid, vanillylmandelic acid and metanephrines. Liver and renal function were normal, with two abdominal ultrasounds confirming normal liver and spleen size and echotexture. Bone marrow analysis indicated normal tri-lineage hematopoiesis with normal maturation, and minimal infiltration by mast cells. Given his elevated tryptase, and diagnosis of an IgE-dependent disease related mast cell disorder, the patient was maintained on cetirizine, and advised to avoid almonds and yellow pepper. He was prescribed epinephrine auto-injectors.

To date, there is one case report of shellfish anaphylaxis associated with mast cell activation syndrome (MCAS). In contrast, there is an established association, and parameters for management, of mast cell disorders associated with venom allergy [stinging insect parameter]. In our patient, it is unclear whether the mast cell disorder was incidentally discovered and secondary to an IgE mediated allergy, or if mast cell dysfunction unmasked sub-clinical food sensitizations. MCAS can predispose to severe anaphylaxis reactions. In adults with newly discovered food allergy, clinicians should have a high suspicion for newly acquired or indolent mast cell disorders, particularly if there is a history of severe cardiovascular compromise.

**Consent:** The patient provided written informed consent for publications of the details in this case report.

### A94 Low dose mepolizumab in the treatment of idiopathic hypereosinophilic syndrome

#### Matthew M. Laird^1^, Ridhu C. Burton^2^

##### ^1^Department of Physiology and Pharmacology, University of Western Ontario, London, Ontario, Canada; ^2^Associate Professor, College of Human Medicine, Michigan State University, Lansing, Michigan, USA

###### **Correspondence:** Matthew M. Laird

*Allergy, Asthma & Clinical Immunology* 2018, **14(Suppl 1):**A94

**Background:** Hypereosinophilic syndrome (HES) is a multisystemic disease characterized by blood eosinophilia (> 1500 eosinophils/mm^3^) without a secondary cause of eosinophilia. Mucocutaneous presentations occur in 25–50% of patients.

**Case study:** An 81-year-old female presented with a 5 year history of chronic pruritus was presented. Failed treatments included: topical steroids, steroid injections, phototherapy, and Goeckerman regimen. Punch skin biopsy: superficial perivascular lymphocytic infiltrate with mild eosinophilia. Travel history negative. No improvement with discontinuation of Benazepril and Simvastatin. CT chest, abdomen and pelvis no lymphadenopathy. Failure on Zyrtec, Hydroxyzine, Ranitidine, Montelukast.

Eosinophil count: 1900 eos/mm^3^, IgE 213. No secondary causes of eosinophilia: Normal: blood smear, tryptase, B12, troponin, ECG. Negative: stool O&P, urine culture, serology Strongyloides, EBV & Hepatitis screen, HIV declined. Bone marrow aspirate and biopsy: no atypical T-cells, no dysplasia in the erythroid, myeloid or megakaryocyte series but presence of increase eosinophils. No evidence for rearrangement of PDGFRA/B or FGFR1.JAK2 V617F or bcr-abl mutation. TCR gene rearrangement studies negative.

Patient treated with high dose steroids but unable to reduce Prednisone to < 20 mg/day. Failed second line treatments included: Hydroxyurea 500 mg, Cyclophosphamide 50 mg. Imatinib mesylate 400 mg which caused retinal hemorrhage. Trial of Mepolizumab 100 mg subcutaneous injections q 4 weeks was initiated, pruritus improved after first dose and Imatinib discontinued. After 3 doses of Mepolizumab, itching stopped, Prednisone dose reduced to 4 mg daily. After 5 doses, Mepolizumab discontinued - patient still symptom free 4 months later with absolute eosinophil count 33 eos/mm^3^.

**Conclusion:** There is no current treatment for HES patients not caused by M-HES or L-HES who have failed first and second line agents. Past trials with high dose mepolizumab 750 mg IV for idiopathic HES showed good response. We report use of Mepolizumab 100 mg subcutaneous q 4 weeks for 5 doses, with complete amelioration of symptoms.

**Consent:** Written informed consent was obtained from the patient for publication of this abstract. A copy of the written consent is available for review by the editor of this journal.

### A95 A report of a 35 year old man newly diagnosed with familial hemophagocytic lymphohistiocytosis type 2 with absent perforin activity

#### Valerie Massey^1^, Imran Ahmad^2^, Hugo Chapdelaine^3^

##### ^1^Internal Medicine Program, University of Montreal, Montreal, Quebec, Canada; ^2^Hematopoietic Cell Transplant Program, Hôpital Maisonneuve-Rosemont, University of Montreal, Montreal, Quebec, Canada; ^3^Allergy and Clinical Immunology Department, Centre Hospitalier de l’Université de Montréal (CHUM), Montreal, Quebec, Canada

###### **Correspondence:** Valerie Massey

*Allergy, Asthma & Clinical Immunology* 2018, **14(Suppl 1):**A95

**Introduction:** Familial hemophagocytic lymphohistiocytosis (FHL) is a life-threatening disease characterized by fever, cytopenias and hepatosplenomegaly. Identifying FHL is crucial as definitive treatment is hemopoietic cell transplantation (HCT). FHL usually presents in toddlers, only few adult cases have been described. Most adult patients have hypomorphic mutation and residual or normal perforin level.

**Case presentation:** At ages 5, 10 and 12, the patient was hospitalized for recurrent episodes of fever, cytopenias, hepatosplenomegaly and encephalitis. He was diagnosed with chronic EBV infection with central nervous system involvement. At age 26 and 35, he presented with cytopenias, fever and splenomegaly. Hemophagocytic lymphohistiocytosis was suspected both times but no macrophage activation was found. He was treated with steroids and IVIG. On flow cytometry, NK cells were low and perforin was absent. Molecular biology showed heterozygosity for the PFR1 gene with P39H and G149S missenses mutations. The patient was diagnosed with FHL type 2 with complete absence of perforin. At age 36, he underwent allogeneic HCT (unrelated donor) after a conditioning regimen including fludarabine, melphalan and alemtuzumab. As of day +217 after HCT there is no sign of relapse or graft-versus-host disease, despite mild neurologic sequelae.

**Discussion:** To our knowledge, we describe the oldest patient with FHL without residual perforin activity. He survived 36 years with presumed complete absence of perforin, thought to be fatal in the first years of life, with a promising 6-month follow-up after HCT. This atypical case presented as a subacute perforinopathy without residual perforin level. Furthermore, missenses mutations are thought to have a milder impact than nonsense mutations on perforin level. The optimal conditioning regimen for HLH in adult is unknown, different regimens have been described. This case emphasizes the need for increasing awareness on the existence of FHL in adults.

**Consent:** Informed written consent was obtained from the patient.

### A96 Progressive myopathy with functional impairment: a potential presentation of late-onset eosinophilic myositis

#### Sanju Mishra^1^, Scott Lee^2^, Volodko Bakowsky^3^

##### ^1^Department of Medicine, Division of Clinical Immunology & Allergy, Western University, London, Ontario, Canada; ^2^Department of Medicine, Dalhousie University, Halifax, Nova Scotia, Canada; ^3^Department of Medicine, Division of Rheumatology, Dalhousie University, Halifax, Nova Scotia, Canada

###### **Correspondence:** Sanju Mishra

*Allergy, Asthma & Clinical Immunology* 2018, **14(Suppl 1):**A96

**Background:** Hypereosinophilic syndrome (HES) is a rare condition that is characterized by peripheral eosinophilia (an absolute eosinophil count (AEC) of > 1.5 × 10^9^/L) for greater than 6 months and end-organ damage with the exclusion of other secondary causes. Eosinophilia with myositis (EWM) consists of peripheral eosinophilia (> 0.5 × 10^9^/L) and eosinophilic muscle infiltrates. We present the case of a patient with late-onset EWM and HES traits with symptoms that have been refractory to glucocorticoids.

**Case presentation:** RM is an 82 year old male who experienced a 4 year history of progressive myopathy, arthralgias and weight loss in the setting of a climbing AEC. RM was referred to the neurology service, due to functional impairment, and was subsequently admitted to hospital to expedite management. Initially, RM had an elevated AEC which normalized with glucocorticoids. However, even with continued steroid use, RM still experiences myositis symptoms and a recent MRI showed active disease involving bilateral proximal thigh muscle groups. Although eosinophilic myositis remains high on the differential, CK levels have always been normal which is unusual in this disease process.

**Discussion:** Investigations have been negative for a clear neurologic, rheumatologic, infectious or hematologic etiology. RM’s IgE level was mildly elevated and a consultation to clinical immunology and allergy is pending. On bone marrow biopsy, a PDGFRa/CHIC2 deletion, a surrogate for the FIP1L1-PDGFRA mutation, was negative, therefore imatinib is likely to be of limited use. Hydroxyurea therapy was therefore initiated and RM will follow-up with the rheumatology service. If symptoms remain refractory, there could be consideration of mepolizumab therapy.

**Conclusion:** The case describes symptoms concerning for eosinophilic myositis with a late age of presentation, and unusual biochemical markers. The case also emphasizes the importance of early recognition of eosinophilia related end-organ damage, and consideration of second-line therapies, in difficult-to-treat eosinophilia with myositis.

**Consent to publish:** Written informed consent to publish was obtained from the patient.

### A97 Wide complex rhythm following intravenous epinephrine injection for venom induced anaphylaxis: a case report

#### Nazanin Montazeri, Samira Jeimy, Harold Kim

##### Department of Clinical Immunology and Allergy, Western University, London, Ontario, Canada

###### **Correspondence:** Nazanin Montazeri

*Allergy, Asthma & Clinical Immunology* 2018, **14(Suppl 1):**A97

**Background:** Epinephrine, the main treatment for anaphylaxis, exerts its effects through alpha and beta-adrenergic receptors. ^1^The cardiac effects are tachycardia and enhanced myocardial contractility. These are mediated through beta-1 adrenergic receptors. ^1^Despite its lifesaving effects, epinephrine has been associated with serious cardiac and non-cardiac adverse effects.^2^

**Case study:** We report the case of a 25-year-old healthy male who was stung on the head by an insect. He developed local swelling, numbness of his lips, urticarial lesions on his wrists, and mild shortness of breath. He came into an emergency room. He was diagnosed with anaphylaxis and inadvertently treated with IV Epinephrine. He received 0.2 mg of 1:1000 Epinephrine pushed intravenously. Within minutes, he reported severe headache, feeling of doom and lightheadedness. The patient was placed on cardiac monitoring and a wide complex rhythm was identified and recorded. His blood pressure was measured at 200/130 mmHg. Within minutes, he spontaneously reverted to sinus rhythm and he clinically recovered. He was referred to an allergist and was treated for yellow-jacket allergy.

**Conclusion:** Serious cardiac adverse effects due to epinephrine have been reported in the past. They include hypertension, ventricular arrhythmias, myocardial infarction and pulmonary edema.^2^ However, to our knowledge this is the first case of a wide complex rhythm due to a very small dose of intravenous epinephrine injection for treatment of anaphylaxis recorded on a rhythm strip. Our case highlights a well-documented episode of a potentially deadly arrhythmia caused by a small dose of IV epinephrine.

**Consent to publish:** Written informed consent to publish was obtained from the patient.

### A98 Intentional poisoning with peanut as a cause of recurrent anaphylaxis

#### Kara Robertson^1^, Harold Kim^2,3^

##### ^1^Department of Medicine, Dalhousie University, Halifax, Nova Scotia, Canada; ^2^Western University, Allergy and Immunology Program, London, Ontario, Canada; ^3^McMaster University, Kitchener, Ontario, Canada

###### **Correspondence:** Kara Robertson

*Allergy, Asthma & Clinical Immunology* 2018, **14(Suppl 1):**A98

**Background:** Anaphylaxis is a serious potentially life-threatening allergic reaction. However, recurrent anaphylaxis is a rare phenomenon since most life-threatening allergies can be avoided with strict allergen avoidance.

**Case report:** A 44 year-old male with a known peanut allergy first presented to our clinic with symptoms of severe recurrent anaphylaxis, requiring epinephrine administration in the emergency room. These episodes had been associated with consumption of food (though he had fastidiously avoided any ingestion of peanuts) at lunchtime, and clinical history did not correspond with objective testing. The following year, he returned to us having experienced a number of recurrent episodes of anaphylaxis in association with the consumption of specific foods at lunchtime. At this point, the differential was broad but investigations did not clearly reveal any organic disease process. He was seen multiple times in the past 4 years concerning at least seven episodes of anaphylaxis. We discussed the possibility of contamination where the patient revealed to us that the police had suggested that his wife might have been contaminating his food. He discovered that his wife, who suffers from bipolar affective disorder, was sprinkling peanut dust on his lunches in an attempt to harm him. She subsequently was admitted to a psychiatric facility where she has remained. On this patient’s most recent allergy testing, he was skin test negative and IgE negative to peanut.

**Conclusion:** This is the first report of intentional poisoning with peanut allergen identified in a case of recurrent anaphylaxis. Since the most recent testing no longer revealed an allergy to peanuts, it is possible that this individual may have resolved his allergy from the repeated exposures. This unusual case reinforces the importance of taking an accurate history, the importance of trusting the results of objective testing, and of having a broad differential diagnosis for the cause of life threatening anaphylactic reactions. Intentional poisoning with food allergens should be considered in recurrent and unexplained anaphylaxis.

**Consent to publish:** Written informed consent to publish was obtained from the patient.

### A99 Omalizumab treatment leading to excessive weight gain

#### Juan C. Ruiz^1^, Stephen Cheuk^2^, Ann Clarke^3^

##### ^1^Department of Medicine, Cumming School of Medicine, Calgary, Alberta, Canada; ^2^Arid Mountain, Clinical Allergist, Calgary, Alberta, Canada; ^3^Division of Rheumatology, Cumming School of Medicine, Calgary, Alberta, Canada

###### **Correspondence:** Juan C Ruiz

*Allergy, Asthma & Clinical Immunology* 2018, **14(Suppl 1):**A99

**Background:** DL is a healthy 32-year-old male with Cold Induced Urticaria (CIU) successfully treated with Omalizumab who experienced severe weight gain. A systematic review of the literature showed that there is only one other case of weight gain induced by Omalizumab in the treatment of severe asthma.

**Case:** DL was started on monthly Omalizumab in August 2015 and gained 22 lb (235–257) over 4 months. The weight gain was gradual, averaging 7 lb per month. Mr. DL’s thyroid, liver, renal function and complete blood count labs were normal. Due to weight gain, he discontinued Omalizumab between December and March 2015 and lost 4 lb (257–253). Due to uncontrolled CIU, he was restarted on Omalizumab every 6 weeks in March 2016 for 4 months. During this time, Mr. DL increased his physical activity and modified his diet, and lost 8 lb over 3 months (253–244). In September 2016, he was re-started on monthly Omalizumab for 7 months due to worsening CIU and experienced an 11-pound weight gain (244–255) despite continued intense exercise and a healthy diet. DL stopped Omalizumab between March and June 2017 and lost 17 lb (255–238) although he stopped exercising and dieting during this time.

**Results:** We observed a trend towards weight loss when Omalizumab was stopped or decreased in frequency.

**Conclusion:** Although there are no manufacturer disclosures regarding weight gain with Omalizumab, we suspect this is an underreported side effect. Our patient experienced severe weight gain while on treatment, which was reversible upon stopping the treatment for a few months. We suspect that Omalizumab may play a role in the endocrine axis of weight regulation in certain patients.

**Consent:** Written informed consent was obtained from the patient for publication of this case report and any accompanying images.

### A100 First reported case in Canada of anaphylaxis to Lupine in a child with peanut allergy

#### Lianne Soller, Edmond S. Chan

##### Division of Allergy and Immunology, Department of Pediatrics, Faculty of Medicine, BC Children’s Hospital, University of British Columbia, Vancouver, British Columbia, Canada

###### **Correspondence:** Lianne Soller

*Allergy, Asthma & Clinical Immunology* 2018, **14(Suppl 1):**A100

**Background:** Lupine (*Lupinis albus*) is a member of the legume family. In Europe, it is often used in baked goods as an alternative to wheat or soy flour [1]. Lupine is recognized as a priority allergen in the European Union because of its cross-reactivity with peanut. However, it has yet to reach the Canadian list, as it hasn’t been present in most Canadian food products.

**Case presentation:** We describe a 10-year old boy with confirmed peanut allergy, who developed anaphylaxis to lupine flour in May 2017. A few minutes after eating pancakes made with PC Blue Menu Buttermilk Protein Pancake Mix that didn’t contain any of his known allergens (peanut, tree nuts), he developed a tingly mouth followed by throat tightness, severe stomach ache, lightheadedness, cough, hoarse throat, stuffy nose, sneezing, and fatigue. He refused EpiPen^®^, but was given Reactine. The symptoms resolved after 3 h, but he was still unwell the following day. In a conversation between the mother and Dr. Chan, it was determined that lupine was likely the cause of the reaction. To confirm, he was brought into clinic for skin testing to lupine. Results were consistent with lupine allergy (pancake mix: 10 × 7 mm, lupine bean: 12 × 6 mm). The family has since reported this to the Canadian Food Inspection Agency, resulting in a consumer advisory bulletin and product recall.

**Conclusion:** This is the first reported case of allergic reaction to lupine in Canada, and highlights the need for education of Canadian families with peanut allergy as well as allergists, regarding the possibility of cross-reactivity between peanut and lupine and its current presence in the Canadian food supply. In addition, a precautionary label for those with peanut allergy who purchase products containing lupine and addition of lupine to the list of priority allergens in Canada should be considered.

**Consent to publish:** Written informed consent to publish was obtained from the patient’s guardians.

### A101 NOD2 and NLRP3 mutation in a patient manifested with chronic periodic urticaria and angioedema

#### Safiah H. Sumayli, Phil Gold

##### Allergy Immunology Section, McGill University, Montreal, Quebec, Canada

###### **Correspondence:** Safiah H. Sumayli

*Allergy, Asthma & Clinical Immunology* 2018, **14(Suppl 1):**A101

**Background:** We are writing about the possible correlation between NOD2 and/or NLRP3 mutations. Disorders in innate immune system are associated with a group of diseases, collectively called autoinflammatory diseases. A group of monogenetic defects were described in association with many of these diseases, such as: MEFV, MVK, TNFR1, NLRP3, NOD2, PSTPIP1 mutations with FMF (familial Mediterranean fever), HIDS (hyper IgD syndrome), TRAPS (Tumor necrosis factor receptor associated Periodic Syndrome), CAPS (Cryopyrin associated autoinflammatory syndromes), Blau syndrome and PAPA (Pyogenic arthritis pyoderma gangrenosum and acne) respectively. A considerable number of patients seen in allergy clinic for urticaria and/or angioedema have accompanying symptoms of nonspecific bone aches, subjective fever or a typical treatment resistant skin involvement.

**Methods:** In our asthmatic 53-year-old female patient who had antihistamine resistant urticaria along with recurrent episodes of facial angioedema, joint stiffness and wheezing, we considered genetic screening for a possible autoinflammatory syndrome although her phenotype is not typical of any of the known causative genetic defects.

**Results:** The test came back positive with heterozygous mutation in the NLRP3 gene and this variant has not previously been reported. Additionally, a heterozygous variant in the NOD2 gene was identified. In conclusion, a newly described variant of previously identified genetic defect was found in a middle-aged female with refractory urticaria and angioedema with associated systemic symptoms.

**Conclusion:** A true genotype–phenotype–correlation cannot be theoretically made unless functional studies of the identified variant are performed.

**Consent to publish:** Written informed consent to publish was obtained from the patient.

### A102 Successful desensitization in a patient with anaphylaxis to 5% human serum albumin

#### Yuanhang Sun^1^, Patricia Pelletier^2^, Phil Gold^1^

##### ^1^Division of Clinical Immunology & Allergy, Department of Medicine, McGill University, Montreal, Quebec, Canada; ^2^Division of Hematology, Department of Medicine, McGill University, Montreal, Quebec, Canada

###### **Correspondence:** Yuanhang Sun

*Allergy, Asthma & Clinical Immunology* 2018, **14(Suppl 1):**A102

**Background**: Human serum albumin (HSA) is a plasma-derived blood product used for a variety of indications, including plasmapheresis. These products are prepared from pooled human plasma by cold ethanol fractionation, heat inactivation, and stabilization with sodium caprylate. Albumin-induced anaphylaxis is rare with a reported incidence of 0.011–0.099%. The few cases which exist have occurred during intraoperative infusions, venom immunotherapy or with plasmapheresis. Other than avoidance, the medical literature offers no solutions for patients with albumin-induced anaphylaxis. Hence, we present a successful desensitization protocol in a patient with albumin-induced anaphylaxis.

**Case presentation**: A 26-year-old woman with a history of focal segmental glomerulosclerosis (FSGS) and renal transplant developed a relapse of her FSGS. Plasmapheresis with 5% HSA was started as treatment but within 2 min of her first session, the patient developed cough, pruritic palms and soles, generalized flushing and abdominal cramps with diarrhea. A second session was attempted 2 days later with the same anaphylactic reaction. Blinded challenge to 5% HSA was positive. Intradermal skin testing of 5% HSA was positive from 1:10 to 1:1000 dilutions. Desensitization to 5% HSA was performed in the intensive care unit. Subsequently she tolerated ten sessions of plasmapheresis with 5% HSA. One month after her last plasmapheresis, re-exposure to 5% HSA produced the same anaphylactic reaction.

**Conclusion:** The mechanisms of albumin-induced anaphylaxis are unclear, and both IgE-mediated and non-immunologic (‘anaphylactoid’) mechanisms have been postulated. Commercial production of HSA leads to albumin aggregates of ≤ 10% of the total protein content which have elicited anaphylactoid reactions in dogs. Caprylate-modified albumin can be immunogenic in humans. In a series published by Ring et al. three patients with anaphylactoid reactions to HSA had positive intradermal skin test to albumin aggregates and caprylate-modified albumin, suggesting the presence of a specific IgE antibody. Given the importance of plasmapheresis for our patient’s underlying FSGS, this case describes a successful desensitization to HSA leading to temporary immune tolerance.

**Consent:** Written informed consent for publication of their clinical details was obtained from the patient. A copy of the consent form is available for review by the Editor of this journal.

### A103 Meningococcal sepsis in possible complement deficiency: case report

#### Herman Tam, Tamar Rubin

##### Department of Pediatrics and Child Health, University of Manitoba, Winnipeg, Manitoba, Canada

###### **Correspondence:** Herman Tam

*Allergy, Asthma & Clinical Immunology* 2018, **14(Suppl 1):**A103

**Introduction:** Complete absence of some complement components confers increased susceptibility to encapsulated bacteria. We present a patient with *Neisseria meningitides* bacteremia, and only mildly abnormal complement studies of uncertain clinical significance.

**Case presentation**: A 13-month-old, previously healthy Métis boy presented with fever, lethargy, and cough. Nasopharyngeal swab was positive for human metapneumonivirus, and blood culture grew *Neisseria meningitides*. He was hospitalized and successfully treated with intravenous antibiotics. One month prior, he received Meningococcal-C conjugate vaccination, in the absence of a known epidemic.

Immunological evaluation repeatedly revealed mildly low CH50 (20.47–29.63 U/mL [41.68–95.06]), with normal C3 and C4 levels. Additional tests sent to a specialized complement lab showed mildly low CH50 (47% [54.8–130.8%]), low AH50 (67 U/mL [77–159]), low C1q level (60.7 mcg/mL [83–125]), low C1r level (51% of standard [61–162]), and low C2 function (7282 U/mL [15,354–46,242]). C1s level, and C5-C9 functions were normal. Immunoglobulin levels and protein vaccine responses were normal. He was vaccinated with Bexsero, Menactra, and Pneumovax. Post-vaccine titres, properdin levels, and ANA are pending.

**Discussion:** Low—but not absent—complement function, and deficiencies of multiple complement components, are typically attributed to consumption, rather than inherited deficiency. Indeed, low CH50 with reduced levels of several early classic complement components in our case may be related to consumption. However, properdin deficiency can predispose to meningococcal infection and may result in only mild AH50 reductions. As well, C2 function in our patient was 50% of the lower limit of normal, possibly attributable to heterozygosity for a defective C2 allele, present in 0.5–1% of Europeans and Ashkenazi Jews respectively, and typically not clinically significant. This case illustrates nuances in complement testing interpretation, and highlights inherited complement deficiencies presenting with low but not absent complement function.

**Consent:** Publication consent was obtained from the patient’s parent.

### A104 An interesting case of beer allergy

#### Kun Tian^1^, Chrystyna Kalicinsky^2^

##### ^1^Department of Medicine, University of Saskatchewan, Saskatoon, Saskatchewan, Canada; ^2^Department of Allergy and Clinical Immunology, University of Manitoba, Winnipeg, Manitoba, Canada

###### **Correspondence:** Kun Tian

*Allergy, Asthma & Clinical Immunology* 2018, **14(Suppl 1):**A104

**Case study:** Beer is one of the most popular beverages and has been consumed all over the globe for centuries. Although many people may have intolerance to beer products, true allergic reaction is rarely seen.

We report a case of a 51 year-old Caucasian man who developed severe immediate hypersensitivity within 10 min of ingesting a bottle of Miller Genuine Draft (MGD) beer while on a boat at a lake. His symptoms were urticaria and laryngeal edema. The reaction was not associated with food co-ingestion, exercise, or insect sting. He denied any additional medication use apart from his regular medications (atorvastatin, aspirin and vitamin D). The patient sought medical attention and was treated acutely with antihistamine, corticosteroid, and epinephrine. His skin prick test was later shown to be positive to MGD at 8 mm, barley at 4 mm, and hops at 4 mm. ImmunoCap was positive to barley at 3 kU/L. His medical history includes a 20-year history of chronic urticaria. The patient believes that in retrospect, each of his previous episodes of urticaria may have been associated with beer ingestion. He only drank Corona beer in the past, which also contains barley and hops.

To our knowledge, our patient is the only Caucasian within the existing literature to be skin test positive to both hops and barley, with corresponding clinical symptoms. It is possible that his chronic urticaria is a manifestation of beer allergy.

**Consent:** A written informed consent was obtained from the patient to publish this case.

### A105 Use of rupatadine in the management of catamenial anaphylaxis

#### Andrew Wong-Pack^1^, Douglas P. Mack^2,3^, Mariam A.Hanna^2,3^

##### ^1^Schulich School of Medicine and Dentistry, Western University, London, Ontario, Canada; ^2^Department of Pediatrics, McMaster University, Hamilton, Ontario, Canada; ^3^Halton Pediatric Allergy, Burlington, Ontario, Canada

###### **Correspondence:** Andrew Wong-Pack

*Allergy, Asthma & Clinical Immunology* 2018, **14(Suppl 1):**A105

**Background:** Catamenial anaphylaxis describes a condition of recurrent multisystem allergic reactions related to menstruation. Several mechanisms of action have been proposed, however, optimal treatment approach remains unclear. Platelet activating factor (PAF) is a mediator of anaphylaxis and serum levels of PAF correlate with the severity of systemic reactions. Rupatadine is a non-sedating antihistamine with anti-PAF effects. We report the first case of Rupatadine as a management agent in an adolescent with suspected catamenial anaphylaxis.

**Case presentation:** A 13-year-old female patient presented with monthly anaphylactic episodes starting 6 months after menstruation. Assessment by endocrinology suggested a trial of leuprolide injections to prevent episodes of anaphylaxis. Side effects of leuprolide were a concern for the family and instead she was started on Rupatadine daily for 2 weeks prior to and during each menses to prevent symptoms. The patient did not experience further episodes of anaphylaxis during their therapeutic trial.

**Discussion:** Experience with potential management of catamenial anaphylaxis includes nonsteroidal anti-inflammatory drugs (NSAIDS) and oral conjugated estrogen to surgical intervention such as bilateral oophorectomy or total abdominal hysterectomy. Treatment options with fewer potential side effects for this population should be considered. A trial of Rupatadine was shown to prevent anaphylaxis in a patient with catamenial anaphylaxis.

**Consent:** Written informed consent was obtained from the patient for publication of this abstract. A copy of the written consent is available for review upon request.

### A106 A case of systemic allergic reaction to breast milk

#### David Yue, Rebecca Pratt

##### McMaster University, Hamilton, Ontario, Canada

###### **Correspondence:** David Yue

*Allergy, Asthma & Clinical Immunology* 2018, **14(Suppl 1):**A106

**Background:** Estimates of between 5–10% of young children are sensitized/allergic to foods. Cow’s milk, hen’s egg, and peanuts are among the most common. As there is currently no cure, strict avoidance is the standard of care. For IgE-mediated food allergies, there are no clear guidelines regarding maternal dietary restriction.

**Case description:** A previously healthy 5-month-old boy was referred regarding severe atopic dermatitis and the possible contribution of food allergy. Atopic dermatitis started at 2-months-old with current diffuse involvement and significant associated pruritis. Thus far, he had not responded to topical corticosteroids and antifungals. Mother had also trialed 2 weeks off dairy with no improvement.

At time of referral, he was exclusively breastfed. Skin-prick testing were positive to milk (9 mm), egg (15 mm), and peanut (4 mm). Mother was counseled regarding strict avoidance for both baby and mother. There was once again no improvement.

Investigations from their dermatologist revealed zinc deficiency and dermatitis responded dramatically to oral zinc supplementation. With no benefit noted with maternal dietary restriction and atopic dermatitis now under control, mother opted to reintroduce milk into her diet. Within 4 days of maternal milk reintroduction, there was acute worsening of his atopic dermatitis, along with emesis and left eye angioedema on two separate occasions.

**Case discussion**: Human milk does not contain beta-lactoglobulin, unlike other mammalian milks including cow’s milk. Transfer of maternally-consumed cow’s milk protein to breastmilk has been previously confirmed. Systemic reactions of food allergens in breastmilk are not common, however there are case reports relating to peanut and fish allergens in breastmilk. Here we described a case of an immediate-type systemic reaction to cow’s milk allergen in breastmilk.

**Conclusion:** Maternally consumed food proteins can be transferred into breastmilk and though rare, this can result in immediate-type systemic reactions to maternally consumed food allergens in breastmilk.

**Consent:** Written consent for publication of information about the patient and his case was obtained from his primary caregiver.

### A107 Subcutaneous immunoglobulin for the treatment of chronic spontaneous urticaria: a case report

#### Karver Zaborniak^1^, Chrystyna Kalicinsky^2^

##### ^1^Department of Internal Medicine, University of Manitoba, Winnipeg, Manitoba, Canada; ^2^Department of Internal Medicine, Allergy and Clinical Immunology, University of Manitoba, Winnipeg, Manitoba, Canada

###### **Correspondence:** Karver Zaborniak

*Allergy, Asthma & Clinical Immunology* 2018, **14(Suppl 1):**A107

**Introduction:** Chronic spontaneous urticaria (CSU) is a debilitating condition, affecting up to 1% of the population. CSU is defined by the presence of urticaria (with or without angioedema), which persists for at least 6 weeks. First-line treatment of CSU includes second-generation H1-antihistamines, followed by up-titration of the antihistamine dose. Refractory treatment options include cyclosporine, omalizumab, and IVIG.

**Case:** A 45-year-old female was diagnosed with CSU, and initially prescribed mesalazine and desloratadine. Due to a lack of clinical response, the desloratadine dose was increased, and montelukast was added, without significant clinical improvement. Once-monthly IVIG was subsequently introduced, and the patient reported improvement for several days after the infusion. The IVIG dose was increased, and the dosing interval shortened, which resulted in remission from pruritus and skin lesions. After 6 months of therapy, the patient developed poor venous access. With patient consent, training was provided for self-administered subcutaneous immunoglobulin (SCIG). In subsequent follow-up, remission was maintained. Reduction of the infusion frequency is currently being considered.

**Discussion and conclusion**: This case serves to demonstrate that SCIG may be an alternative and effective treatment in cases of refractory CSU. Clinical data has begun to establish the effectiveness of IVIG for CSU. However, hospital-based administration remains a burden for both patients and the healthcare system. With further clinical study, a self-administered SCIG regimen may prove to be a solution for both the cost to our healthcare system, and the patients within it.

**Consent:** Written informed consent to publish has been obtained, and provided to CSACI.

